# ESS2 controls prostate cancer progression through recruitment of chromodomain helicase DNA binding protein 1

**DOI:** 10.1038/s41598-023-39626-0

**Published:** 2023-07-31

**Authors:** Sayuri Takahashi, Ichiro Takada, Kenichi Hashimoto, Atsushi Yokoyama, Tohru Nakagawa, Makoto Makishima, Haruki Kume

**Affiliations:** 1 Department of Urology, The Institute of Medical Science, The University of Tokyo, 4-6-1 Shirokanedai, Minato-Ku, Tokyo, 108-8639 Japan; 2 Department of Urology, The Faculty of Medicine, The University of Tokyo, 7-3-1 Hongo, Bunkyo-Ku, Tokyo, 113-8655 Japan; 3 Division of Biochemistry, Department of Biomedical Sciences, School of Medicine, Nihon University, Itabashi-Ku, Tokyo, 173-8610 Japan; 4 Department of Molecular Endocrinology, Tohoku University Graduate School of Medicine, 2‐1 Seiryo‐machi, Aoba‐ku, Sendai, Miyagi 980‐8575 Japan; 5 Department of Urology, Teikyo University School of Medicine, 2-11-1 Kaga, Itabashi-Ku, Tokyo, 173-8605 Japan

**Keywords:** Cancer, Cell biology, Oncology

## Abstract

Molecular targeted therapy using poly (ADP-ribose) polymerase inhibitors has improved survival in patients with castration-resistant prostate cancer (CRPC). However, this approach is only effective in patients with specific genetic mutations, and additional drug discovery targeting epigenetic modulators is required. Here, we evaluated the involvement of the transcriptional coregulator ESS2 in prostate cancer. ESS2-knockdown PC3 cells dramatically inhibited proliferation in tumor xenografts in nude mice. Microarray analysis revealed that ESS2 regulated mRNA levels of chromodomain helicase DNA binding protein 1 (*CHD1*)-related genes and other cancer-related genes, such as *PPAR-γ*, *WNT5A*, and *TGF-β,* in prostate cancer. ESS2 knockdown reduced nuclear factor (NF)-κB/CHD1 recruitment and histone H3K36me3 levels on the promoters of target genes (*TNF* and *CCL2*). In addition, we found that the transcriptional activities of NF-κB, NFAT and SMAD2/3 were enhanced by ESS2. Tamoxifen-inducible Ess2-knockout mice showed delayed prostate development with hypoplasia and disruption of luminal cells in the ventral prostate. Overall, these findings identified ESS2 acts as a transcriptional coregulator in prostate cancer and ESS2 can be novel epigenetic therapeutic target for CRPC.

## Introduction

Prostate cancer causes significant mortality among men world wide^1,2^. The 5-year survival rate for men with prostate cancer who develop metastatic disease is only 29%^1^. Patients with metastatic castration-resistant prostate cancer (CRPC) have several treatment options, including taxanes, androgen-signaling-targeted inhibitors, and bone-targeted radiopharmaceutical agents^3^; however, these therapies are associated with toxicity and a limited durable response^4^. The inhibitory effects of the poly (ADP-ribose) polymerase (PARP) inhibitor olaparib on CRPC metastasis were confirmed in phase III clinical trials^5,6^. PARP repairs single-strand DNA breaks through base excision repair pathways; thus, PARP inhibitors increase the number of single-strand breaks and promote cell death^4^. Normal cells with a functional homologous recombination (HR) pathway can repair DNA breaks, even in the presence of olaparib; however, HR-deficient tumor cells, such as those with BRCA1/2 mutations or ATM loss, are unable to be repaired following Olaparib treatment^4^. Notably, few patients have alterations in target genes^6^. Therefore, identification of novel epigenetic therapeutic targets for patients with CRPC, regardless of genetic mutation status, is urgently needed.

Recent genome-wide analyses have identified critical regulators of prostate cancer proliferation and invasion^7,8^. Among these factors, several transcriptional coregulators have been shown to regulate prostate cancer proliferation by interacting with transcription factors that modulate mRNA expression. Transcriptional coregulators do not bind to specific DNA sequences but regulate target gene mRNA expression by associating with transcription factors. This class of proteins includes histone modifiers, chromatin remodeling factors, RNA polymerase regulators, and other proteins^9^. In particular, chromatin remodeling factors have important roles in CRPC^10^, such as chromodomain helicase DNA-binding proteins (CHDs), which are involved in cancer progression^11^.

Ess-2 splicing factor homolog (ESS2; also known as DGCR14), a novel transcriptional coregulator localized in the nucleus^12^, interacts with the nuclear receptor RAR-related orphan receptor gamma/gammat (ROR*γ*/*γ*t), a critical regulatory factor for Th17 cell differentiation^13^ and CRPC via targeting the androgen receptor^14^. ESS2 enhances the transcriptional activity of RORγ/γt in T cells by interacting with bromodomain adjacent to zinc finger domain 1B (BAZ1B) and ribosomal S6 kinase 2 (RSK2)^12^. BAZ1B is a chromatin remodeling factor that regulates transcription, DNA repair, and replication^15^. RSK2 is also an important regulator of prostate cancer proliferation^16,17^. Interactions among ESS2, BAZ1B, and RSK2 may mediate chromatin organization in cancer. Other groups have shown that ESS2 is involved in the splicing C complex^18^, although the role of ESS2 in splicing remains unclear. We have recently found that ESS2 protein associates with the spliceosome complex at the C-terminus and with transcription factors at the N-terminus^19^.

Interestingly, Protein Atlas data (https://v15.proteinatlas.org/ENSG00000100056-DGCR14/cancer) have revealed high expression levels of ESS2 protein in the normal prostate (Supplementary Fig. 1a). ESS2 is also highly and frequently expressed in cancer cells, including prostate cancer cells (Supplementary Fig. 1b,c). Moreover, CD4-specific ESS2-knockout mice show reduced numbers of naïve T cells ^20^, suggesting a role in regulating cell survival. These results indicate that ESS2 may modulate cell proliferation and maintenance in prostate cancer. The early embryonic lethality of Ess2KO mice and the Ess2-dependent regulation of Myc transcriptional activities in naive T cells suggest that Ess2 exerts important functions in stem cells. Based on these results and ESS2 expression in cancer tissues from Protein Atlas data, we hypothesized that Ess2 might also play an important role in cancer cells.

In this study, we aimed to elucidate the role of ESS2 in prostate cancer. To this end, we established ESS2-knockdown PC3 prostate cancer cells and found that these cells showed reduced proliferation accompanied by aberrant mRNA expression of nuclear factor (NF)-κB/CHD1 and prostate cancer-related genes. Our results demonstrated that ESS2 was a critical regulator of prostate cancer proliferation. Overall, ESS2, which is highly and frequently expressed in cancer tissue, may be a candidate of molecular target therapies for CRPC.

## Results

### ESS2 promoted cell proliferation and tumor growth in PC3 cells

**Figure 1:**
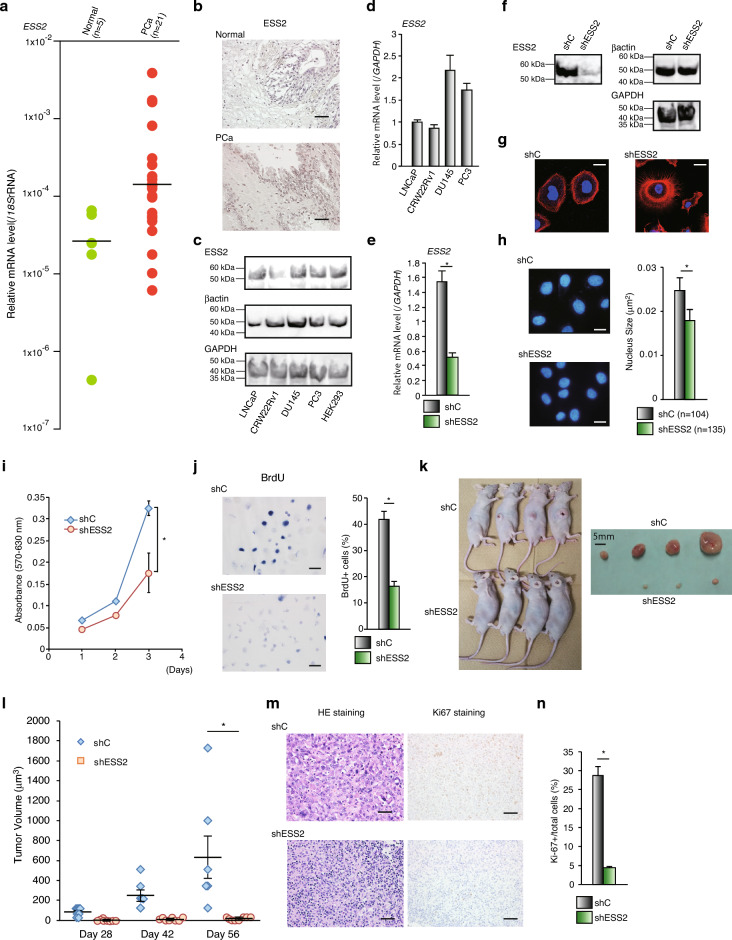
ESS2 regulated the proliferation and tumor formation of PC3 cells. (**a**) RT-qPCR of ESS2 in human normal prostate (Normal) and human prostate cancer (PCa) tissues normalized to the levels of 18SrRNA expression. (**b**) Representative immunostaining of ESS in Normal and PCa. bar = 20 μm. (**c**) Western blotting of ESS2, β-actin and GAPDH in prostate cancer cell lines. Raw data was shown in Supplementary Fig. 7a-c. (**d**) mRNA expression levels of *ESS2* in the indicated prostate cancer cell lines. (**e**) RT-qPCR of *ESS2* in negative control-shRNA (shC)- or ESS2-shRNA (shESS2)-transfected PC3 cells normalized to the level of *GAPDH* mRNA expression. *, *p* < 0.05, Student’s t-test. (**f**) Western blotting of ESS2, β-actin and GAPDH in PC3-shC (shC) and PC3-shESS2 (shESS2) cells. Raw data was shown in Supplementary Fig. 7a-c. (**g**) PC3-shC and PC3-shESS2 cells stained with rhodamine phalloidin (actin polymerization; red) and DAPI (nucleus; blue). Scale bar = 20 μm. (**h**) Representative nuclear staining of PC3-shC (shC) and PC3-shESS2 (shESS2) with DAPI. The difference in the size of the nuclei by DAPI staining was measured using ImageJ (right panel). Scale bar = 20 μm. *, *p* < 0.05, Student’s t-test. (**i**) MTT assays in PC3-shC (shC) and PC3-shESS2 (shESS2) cells. n = 3. *, *p* < 0.05, Student’s t-test. (**j**) Representative BrdU staining (blue) in PC3-shC (shC) and PC3-shESS2 (shESS2) cells. The ratio of BrdU^+^ cells was measured in 5–6 independent areas (right panel). *, *p* < 0.05, Student’s t-test. Scale bar = 50 μm. (**k**) Xenografts of aggregated PC3-shC cells or PC3-shESS2 cells in nude mice (left panel). After 60 days, mice were sacrificed; the extracted tumors are shown in the right panel. (**l**) Tumor growth of xenografts generated from PC3-shC and PC3-shESS2 cells. n = 8. *, *p* < 0.05, Student’s t-test. (**m**) Left and right panels: HE staining (red and blue) and Ki-67 staining (brown) in xenografts derived from PC3-shC and PC3-shESS2 cells. The sections were counterstained with hematoxylin. Scale bar = 50 μm. (**n**) The number of Ki-67-positive cells in xenograft tumors. We calculated results from 7 independent panels for each tumor. *, *p* < 0.05, Student’s t-tests. For RT-qPCR, each experiment was performed at least three times, and the results are presented as means ± standard deviations.

We examined *ESS2* mRNA expression levels in formalin-fixed paraffin-embedded (FFPE) human normal prostate (Normal) and human prostate cancer (PCa) tissues (n = 5 and 21, respectively). *ESS2* mRNA levels were upregulated in prostate cancer (median: Normal = 2.39 × 10^–5^, PCa = 12.8 × 10^–5^; Fig. 1a). Immunostaining of ESS2 also showed that ESS2 highly expresses in PCa tissues (Fig. 1b). Furthermore, we examined ESS2 protein levels in prostate cancer cell lines (LNCaP, CRW22Rv1, DU145 and PC3) and ESS2 highly expressing cells (HEK293 cells) by western blotting. As shown in Fig. 1c, ESS2 protein was highly expressed in LNCaP, DU145, and PC3 cells. Immunofluorescence staining showed that ESS2 protein was highly expressed in androgen-independent prostate cancer cell lines (DU145 and PC3, Supplementary Fig. 2). Moreover, *ESS2* mRNA levels were significantly high in these two cell lines (Fig. 1d). These results showed that ESS2 was highly expressed in androgen-independent prostate cancer cells. Our previous studies showed that ESS2 does not regulate the ligand-dependent transcriptional activity of the androgen receptor (data not shown). Thus, these results suggest that ESS2 has a pivotal role in androgen-independent prostate cancer cells. In particular, we focused on PC3 cells, which are derived from bone metastasis of prostate cancer and show characteristics of small cell neuroendocrine carcinoma (e.g., expressing markers such as chromogranin A, neuron-specific enolase, and the stem cell-associated marker CD44)^21^. In addition, PC3 cells exhibit high metastatic potential compared with DU145 cells.

We generated PC3 cells stably expressing ESS2 shRNA (Fig. 1e,f). Interestingly, PC3-shESS2 cells show aberrant actin polymerization compared with PC3-shC cells (Fig. 1g). Moreover, the nucleus size of PC3-shESS2 cells was smaller compared with that of PC3-shC cells (Fig. 1h). These results demonstrated that ESS2 also regulated cell chape and nucleus size. Next, we performed MTT assays to measure the viability of PC-shC and PC-shESS2 cells. PC3-shESS2 cells showed significantly reduced cell proliferation (Fig. 1i) with decreased BrdU incorporation (Fig. 1j). These results demonstrated that ESS2 regulated PC3 cell proliferation.

Next, we transplanted aggregated PC3-shC and PC3-shESS2 cells into nude mice and observed cancer growth. Surprisingly, PC-shESS2 cells showed reduced tumor growth compared with PC-shC cells (Fig. 1k,l). Ki-67 staining of paraffin-embedded tissue sections showed low Ki-67 expression in PC-shESS2 cells transplanted into nude mice (Fig. 1m,n). These results clearly showed that ESS2 controlled prostate cancer proliferation in vitro and in vivo. However, the expression levels of cell-cycle related genes (e.g., *p21* and *p27*) were not correlated with cell growth inhibition in PC3-shESS2 cells (Supplementary Fig. 3).

### ESS2 knockdown regulated gene expression in PC-3 cells

**Figure 2:**
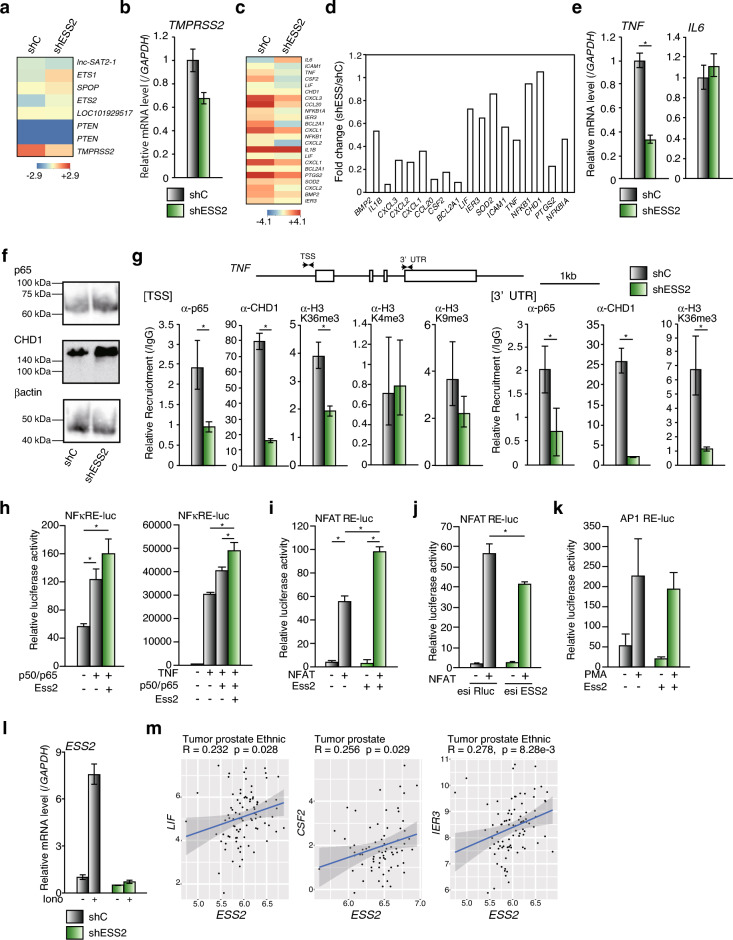
ESS2 regulated NF-κB/CHD1 target genes. (**a**) Heatmap data of prostate cancer-related genes by microarray analysis in PC3-shC and PC3-shESS2 cells. (**b**) RT-qPCR of *TMPRSS2* in PC3-shC and PC3-shESS2 cells normalized to the level of *GAPDH* mRNA. *, *p* < 0.05, Student’s t-test. (**c**) Heatmap data of NF-κB/CHD1 target genes by microarray analysis in PC3-shC and PC3-shESS2 cells. (**d**) Fold changes in gene expression levels (PC3-shESS2/PC3-shC) in (**c**). (**e**) RT-qPCR of *TNF* and *IL6* in PC3-shC and PC3-shESS2 cells normalized to the level of *GAPDH* mRNA. *, *p* < 0.05, Student’s t-test. (**f**) Western blotting of p65, CHD1, and β-actin in PC3-shC and PC3-shESS2 cells. Raw data was shown in Supplementary Fig. 8. (**g**) ChIP-qPCR analysis of *TNF* at the TSS site (TSS) and 3′-UTR site (3′-UTR) with anti-p65, anti-CHD1, and anti-histoneH3K36 trimethylation antibodies in PC3-shC and PC3-shESS2 cells normalized to the input level. *, *p* < 0.05, Student’s t-test. (**h**) Luciferase reporter assays using a p65/p50 expression vectors and/or recombinant TNFα to activate NF-κB on NF-κB response element (NFκRE)-luc reporter vector in HEK293 cells. (**i**) Luciferase reporter assays using a NFAT expression vector to activate NFAT on NFAT response element (NFAT RE)-luc reporter vector in HEK293 cells. (**j**) Luciferase reporter assays using 1 μM phorbol 12-myristate 13-acetate (PMA) to activate AP-1 on AP1 response element (AP1 RE)-luc reporter vector in HEK293 cells. (**k**) ESS2 knockdown by esiRNA decreased the transcriptional activities of NFAT in HEK293 cells. (**l**) RT-qPCR of ESS2 in PC3-shC and PC3-shESS2 cells treated with/without 1 μM ionomycin for 24 h, normalized to the level of *GAPDH* mRNA. *, *p* < 0.05, Student’s t-test. (**m**) Scatter plots showing correlations between ESS2 and NF-κB/CHD1 target gene expression (*IER3*, *LIF*, and *CSF2*) in patients with prostate cancer. For RT-qPCR, ChIP-qPCR and luciferase reporter assays, each experiment was performed at least three times, and the results are presented as means ± standard deviations.

To elucidate the mechanism through which ESS2 affected prostate cancer proliferation, we performed microarray analysis of PC3-shC and PC3-shESS2 cells (Supplementary Fig. 4a). Notably, 2,512 genes showed more than twofold differences in expression in PC3-shC and PC3-shESS2 cells. Among these genes, 1,377 showed downregulation in PC3-shESS2 cells compared with PC3-shC cells (Supplementary Fig. 4b). Kyoto Encyclopedia of Genes and Genomes (KEGG) analysis of cell cycle-related genes did not show any significant differences (Supplementary Fig. 4c). In a comparison of gene sets related to prostate cancer progression (Fig. 2a), transmembrane serine protease 2 expression was reduced in PC3-shESS2 cells (Fig. 2b).

Recently, the chromatin helicase DNA-binding factor CHD1 has been shown to regulate cell proliferation through the NF-κB pathway in prostate cancer cells without phosphatase and tensin homolog (PTEN) expression^22^. Since ESS2 associates with the chromatin remodeling factor BAZ1B^12^, there arises the possibility of ESS2 regulating chromatin remodeling factors in prostate cancer cells. Therefore, we investigated genes regulated by CHD1 using microarray analysis and found that the expression levels of NF-κB/CHD1 target genes were reduced in PC3-shESS2 cells (Fig. 2c,d), despite a lack of change in CHD1 expression. We also confirmed the reduction in tumor necrosis factor (*TNF*) mRNA levels by reverse transcription quantitative polymerase chain reaction (RT-qPCR), whereas interleukin-6 expression was not changed in PC3-shESS2 cells (Fig. 2e). These results suggested that not all CHD1 target genes were regulated by ESS2.

Although protein levels of p65 were unchanged and CHD1 were slightly increased (Fig. 2f), chromatin immunoprecipitation (ChIP)-qPCR showed that p65 and CHD1 recruitment decreased significantly on the *TNF* promoter and 3′-untranslated region (UTR) in PC3-shESS2 cells (Fig. 2g). We examined the recruitment of several modified histones by ChIP and found that histone H3K36 trimethylation also decreased in PC3-shESS2 cells (Fig. 2g). In contrast, histone H3K4 trimethylation and H3K9 trimethylation on the *TNF* gene were not significantly correlated between PC3-shC and PC3-shESS2 cells. These results clearly showed that ESS2 controlled NF-κB(p65)/CHD1 recruitment to target genes. Moreover, ESS2 overexpression enhanced transcriptional activation of NF-kB (Fig. 2h).

On the *TNF* promoter, NFAT and AP-1 binding sites have been identified^23^. To study these relationships, we performed luciferase reporter assays in HEK293 cells. We used NFAT expression vector to activate NFAT on NFAT response element (NFAT RE)-luc and phorbol 12-myristate 13-acetate (PMA) to activate AP-1 on AP1 response element (AP1 RE)-luc. Interestingly, ESS2 significantly enhanced the transcriptional activities of NFAT (Fig. 2i). In addition, ESS2 knockdown by endoribonuclease-prepared siRNA (esiRNA) (Fig. 2j and Supplementary Fig. 5) decreased the transcriptional activities of NFAT. However, ESS2 overexpression does not enhance transcriptional activities of AP-1 (Fig. 2k). Interestingly, ionomycin (NFAT activator) induced *ESS2* mRNA levels in PC3-shC cells (Fig. 2l). These results show that NFAT regulates mRNA levels of ESS2 in PC3 cells.

Next, we evaluated correlations between ESS2 and CHD1 target gene expression in patients with prostate cancer using the R2 database. Among CHD1 target genes, immediate early response 3 (*IER3*), leukemia inhibitory factor (*LIF*), and colony stimulating factor 2 (*CSF2*) were significantly correlated with ESS2 expression in patients with prostate cancer (Fig. 2m). These results also showed that ESS2 depletion in prostate cancer selectively suppressed CHD1 function.

Overall, our data show that the regulation of transcriptional activity by ESS2 targets a large number of transcription factors and illustrates the complex mechanisms of ESS2 function.

### ESS2 regulated the expression of type I interferon (IFN) response genes

**Figure 3:**
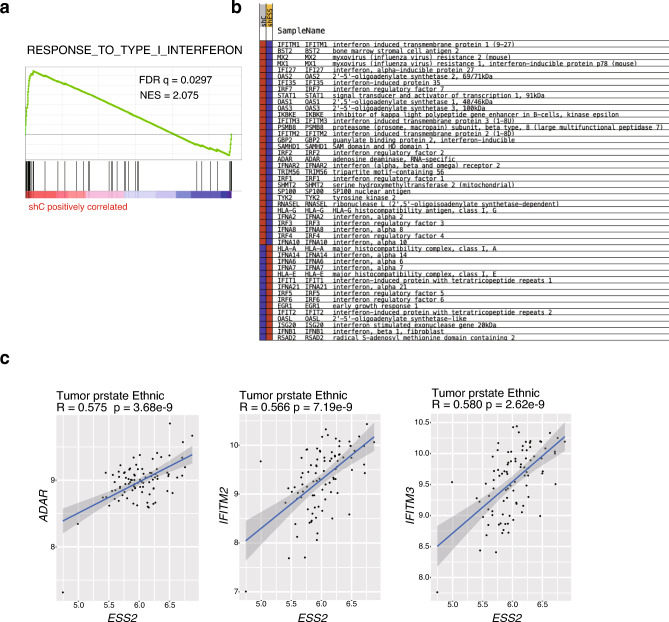
ESS2 knockdown was correlated with type I IFN target genes. (**a**) GSEA analysis comparing PC3-shC and PC3-shESS2 cells. (**b**) Results of genes listed in (**a**). (**c**) Scatter plots showing correlations between ESS2 and type I IFN target gene expression (*ADAR*, *IFTIM2*, and *IFTIM3*) in patients with prostate cancer.

To further elucidate the genome-wide regulation of ESS2 expression, we subjected the microarray results to gene set enrichment analysis (GSEA). We found that only the response to the type I IFN gene set was significantly correlated with the expression changes regulated by ESS2 knockdown (Fig. 3a,b). Moreover, ESS2 expression was highly correlated with adenosine deaminase acting on RNA (*ADAR*), IFN-inducible transmembrane protein (*IFITM*) 2, and *IFITM3* in patients with prostate cancer (Fig. 3c). ADAR protein is a regulatory enzyme for RNA editing and sequestering of noncoding RNA sequences, such as introns and untranslated mRNAs^24^. In prostate cancer cells, ADAR1 mediates the formation of prune homolog 2 with BCH domain (PRUNE2)/prostate cancer antigen 3 (PCA3) double-stranded RNA by regulating PRUNE2 and PCA3 levels via adenosine-to-inosine RNA editing^25^. IFITM3 is a member of the *IFITM* gene family that functions in immune cell signaling, cell adhesion, and stem cell migration^26^. The expression of IFITM3 is positively correlated with Gleason score and T stage, and IFITM3 knockdown inhibits tumor cell migration and invasion; this inhibitory effect was more pronounced in transforming growth factor beta 1 (TGF-β1) pathway-activated cells^27^. Although the inhibitory effects of IFN-α and -β on prostate cancer are milder than those of IFN-γ^28^, these results suggested that ESS2-dependent type I IFN target genes may regulate prostate cancer progression.

### ESS2 regulated peroxisome proliferator-activated receptor (PPAR)-γ and vitamin D receptor (VDR) expression in PC3 cells

Unexpectedly, PC3-shESS2 cells showed aberrant expression levels of only a few gene sets in microarray analysis. By contrast, CD4^+^ T cells from Ess2-knockout mice show aberrant expression related to metabolism and immune diseases (GEO dataset: PRJNA575280). Therefore, we examined the expression of genes that regulate prostate cancer progression. First, we focused on nuclear receptors (NRs), which are fat-soluble ligand-dependent transcription factors. There are 48 homologous NR genes in humans^29,30^, and ESS2 acts as a transcriptional co-activator for one of these NRs (*RORγ/γt*)^12^. NRs have a wide range of functions, including cell proliferation, metabolism, immune system function, and development^31^. In addition to the androgen receptor, other NRs have been reported to contribute to prostate cancer proliferation and progression^32^.

**Figure 4:**
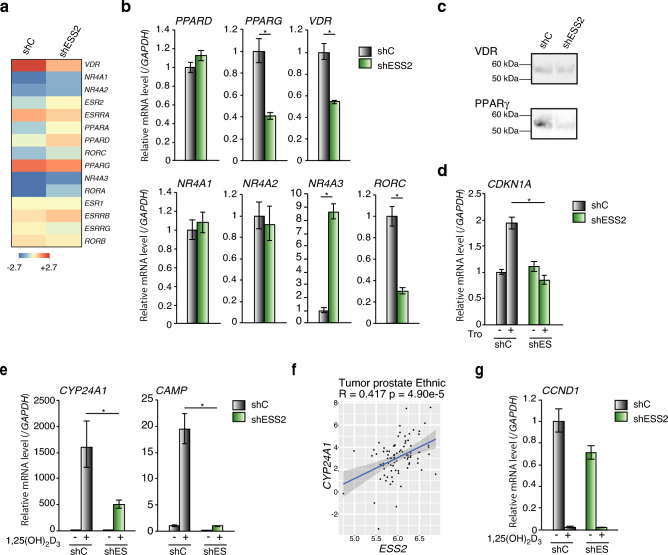
ESS2 regulated *VDR* and *PPAR-γ* mRNA levels. (**a**) Heatmap data of prostate cancer-related NR mRNAs by microarray in PC3-shC and PC3-shESS2 cells. (**b**) RT-qPCR of NR mRNAs in PC3-shC and PC3-shESS2 cells normalized to the level of *GAPDH* mRNA. *, *p* < 0.05, Student’s t-test. (**c**) Western blotting of VDR, PPAR-γ in PC3-shC and PC3-shESS2 cells. Used samples were same as Fig. 1f and control panels (β-actin and GAPDH) are shown in Fig. 1f. Raw data was shown in Supplementary Fig. 8. (**d**) RT-qPCR of the troglitazone-dependent expression of *CDKN1A* mRNA in PC3-shC and PC3-shESS2 cells normalized to the level of *GAPDH* mRNA. *, *p* < 0.05, Student’s t-test. (**e**) RT-qPCR of the 1,25(OH)_2_D_3_-dependent expression of *CY24A1* and *CAMP* in PC3-shC and PC3-shESS2 cells normalized to the level of *GAPDH* mRNA. *, *p* < 0.05, Student’s t-test. (**f**) Scatter plots showing correlations of ESS2 and CYP24A1 expression in patients with prostate cancer. (**g**) RT-qPCR of the 1,25(OH)_2_D_3_-dependent expression of cyclin D1 in PC3-shC and PC3-shESS2 cells normalized to the level of *GAPDH* mRNA. *, *p* < 0.05, Student’s t-test. For RT-qPCR, each experiment was performed at least three times, and the results are presented as means ± standard deviations.

Therefore, we next analyzed the expression levels of prostate cancer-related NRs in PC3-shC and PC3-shESS2 cells using microarray data (Fig. 4a). Unexpectedly, some NRs, such as *VDR* and *PPAR-γ, *were downregulated in PC3-shESS2 cells. In prostate cancer, VDR knockdown induces cell apoptosis and inhibits cell proliferation and tumor growth in immune-incompetent nude mice^33^. PPAR-γ regulates adipocyte differentiation and is a key factor in type II diabetes^34^. PPAR-γ ligands have applications in cancer therapy^35,36^. In androgen-insensitive PC3 cells, PPAR-γ ligands induce p21 and suppress cell proliferation^37,38^.

To verify the microarray data, we performed RT-qPCR and found that mRNA and protein levels of PPAR-γ and VDR were downregulated in PC3-shESS2 cells (Fig. 4b,c). Moreover, PPAR-γ ligand (troglitazone)-dependent *p21* mRNA induction was abrogated in PC3-shESS2 cells (Fig. 4d).

Interestingly, 1α,25(OH)_2_D_3_-dependent induction of VDR target genes (cytochrome P450 family 24 subfamily A member 1 [*CYP24A1*]^33^ and cathelicidin antimicrobial peptide [*CAMP*]^39^) was significantly downregulated in PC3-shESS2 cells (Fig. 4e). We also observed correlations between ESS2 and CYP24A1 expression in patients with prostate cancer (Fig. 4f). However, 1α,25(OH)_2_D_3_-dependent suppression of cyclin D1 mRNA expression^40^ was not abrogated in PC3-shESS2 cells (Fig. 4g).

These results showed that ESS2 regulated prostate cancer proliferation and metabolism by modulating the expression of some NRs, including VDR and PPAR-γ.

### ESS2 regulated C–C motif chemokine ligand 2 (CCL2), WNT5A, and TGFβ1 mRNA levels

**Figure 5:**
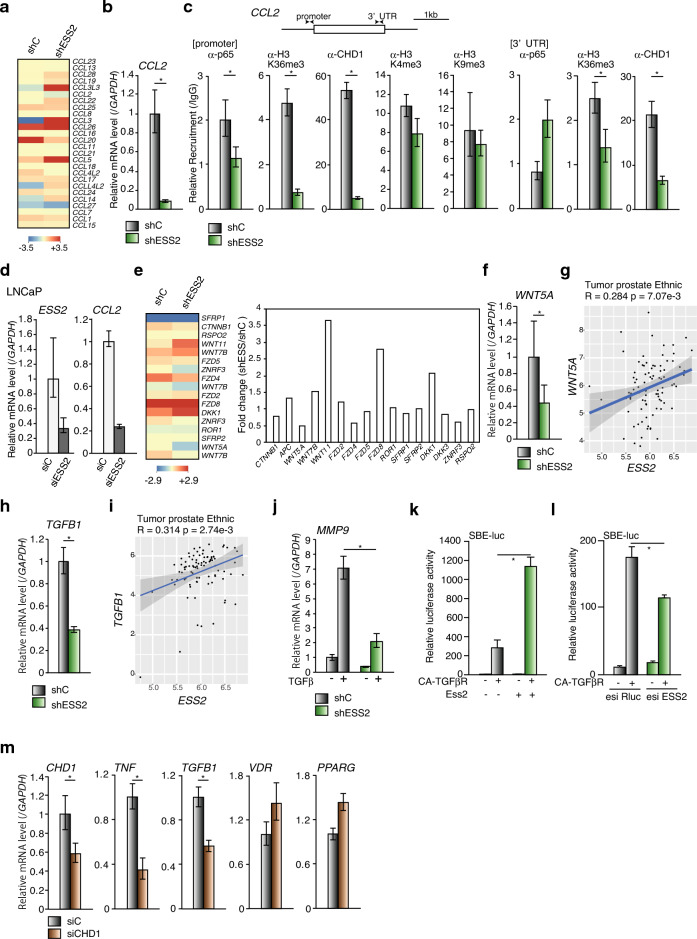
ESS2 regulated *CCL2*, *WNT5A*, and *TGFB1* mRNA levels. (**a**) Heatmap data of CCL mRNAs by microarray in PC3-shC and PC3-shESS2 cells. (**b**) RT-qPCR of *CLL2* mRNA in PC3-shC and PC3-shESS2 cells normalized to the level of *GAPDH* mRNA. *, *p* < 0.05, Student’s t-test. (**c**) ChIP-qPCR analysis of the *CCL2* gene at the TSS site (TSS) and 3′-UTR site (3′-UTR) with anti-p65, anti-CHD1, and anti-histoneH3K36 trimethylation antibodies in PC3-shC and PC3-shESS2 cells normalized to the input level. *, *p* < 0.05, Student’s t-test. (**d**) RT-qPCR of *CCL2* in siC- or siESS2-transfected LNCaP cells normalized to the level of *GAPDH* mRNA. *, *p* < 0.05, Student’s t-test. (**e**) Left panel: heatmap data for WNT-related genes in PC3-shC and PC3-shESS2 cells. Right panel: fold changes in gene expression levels (PC3shESS2/PC3-shC) in microarray data. (**f**) RT-qPCR of *WNT5A* in PC3-shC and PC3-shESS2 cells. *, *p* < 0.05, Student’s t-test. (**g**) Scatter plots showing correlations of ESS2 and WNT5A expression in patients with prostate cancer. (**h**) RT-qPCR of *TGFB1* in PC3-shC and PC3-shESS2 cells. (**i**) Scatter plots showing correlations of ESS2 and TGFB1 expression in patients with prostate cancer. (**j**) RT-qPCR of *MMP9* mRNA in PC3-shC and PC3-shESS2 cells with/without TGFβ normalized to the level of *GAPDH* mRNA. (**k**) Luciferase reporter assays using a constitutively active TGFβR1 (CA-TGFβR) expression vector to activate SMAD2/3 on Smad binding element (SBE)-luc reporter vector in HEK293 cells. (**l**) ESS2 knockdown by esiRNA decreased the transcriptional activities of SMAD2/ in HEK293 cells. *, *p* < 0.05, Student’s t-test. (**m**) RT-qPCR of *CHD1*, *TNF*, *TGFB1, VDR* and *PPARG* in PC3 cells transfected with control siRNA (siC) or CHD1 siRNA(siCHD1)*,* normalized to the levels of *GAPDH* mRNA. *, *p* < 0.05, Student’s t-test. For RT-qPCR, ChIP-qPCR and luciferase reporter assays, each experiment was performed at least three times, and the results are presented as means ± standard deviations.

We further analyzed expression levels of other gene sets in PC3-shC and PC3-shESS2 cells (Fig. 5a,e, and Supplementary Fig. 6). First, we focused on the CCL family, which is associated with cancer progression and metastasis^41^. Interestingly, ESS2 depletion suppressed *CCL2* mRNA expression (Fig. 5a,b). CCL2 (also known as monocyte chemoattractant protein-1) is a member of the CC chemokine family and promotes monocyte chemotaxis to sites of inflammation. In tumors, CCL2 is produced by cancer cells, and multiple transcription factors, including NF-κB, regulate *CCL2* mRNA expression^42^. In PC3 cells, CCL2 acts as a potent chemoattractant and protects against autophagic death through the phosphatidylinositol 3-kinase/AKT pathway^43,44^. Therefore, we next examined p65 and CHD1 recruitment and histone H3K36 trimethylation on the *CCL2* locus using ChIP-qPCR (Fig. 5c). Notably, p65 and CHD1 recruitment on the *CLL2* promoter and 3′-UTR was decreased in PC3-shESS2 cells (Fig. 5c). Histone H3K36me3 levels were also decreased on the *CCL2* promoter and 3′-UTR in PC3-shESS2 cells (Fig. 5c). Interestingly, such ESS2-dependent suppression of *CCL2* mRNA was also observed in LNCaP cells transiently transfected with ESS2 siRNA (Fig. 5d). These results suggested that ESS2 affected prostate cancer progression in vivo by regulating CCL2 expression.

Next, we investigated WNT-related gene expression (Fig. 5e, left). The WNT signaling pathway plays pivotal roles in prostate cancer development, and several Wnt signaling inhibitors have been tested in phase I trials for prostate cancer therapy^45^. Interestingly, among WNT pathway molecules, WNT5A showed reduced expression in PC3-shESS2 cells (Fig. 5e, right). Furthermore, RT-qPCR showed that *WNT5A* mRNA levels were decreased in PC3-shESS2 cells (Fig. 5f), and a correlation was observed between ESS2 and WNT5A expression in patients with prostate cancer (Fig. 5g).

WNT5A is upregulated in prostate cancer and can promote tumor cell invasion through FZD2 and ROR2^46^. In a mouse model of prostate cancer, WNT5A haploinsufficiency prevented the early onset and early lethality of prostate tumors^47^. *WNT5A* mRNA is found in circulating tumor cells from patients with CRPC^48^ and from patients with prostate cancer whose disease progressed while they were undergoing treatment with the androgen receptor inhibitor enzalutamide^8^. Thus, our results showed that ESS2-dependent WNT5A expression may affect prostate cancer progression.

We then investigated epithelial-mesenchymal transition (EMT)-related genes in PC3-shC and PC3-shESS2 cells. The EMT is a characteristic of cancer cell invasion and metastasis and is closely associated with many cancers^49^. In prostate cancer, EMT-related genes regulate metastasis and progression^50^. TGF-β induces the EMT, conferring epithelial tumor cells with aggressive mesenchymal-like phenotypes accompanied by alterations in the expression of intercellular adhesion molecules (such as E-cadherin and N-cadherin) and the secretion of metalloproteinases (MMPs, such as MMP-9), resulting in metastasis. Interestingly, *TGF-β1* mRNA levels were downregulated in PC3-shESS2 cells (Fig. 5h), and ESS2 and TGF-β1 expression levels were significantly correlated in patients with prostate cancer (Fig. 5i).

TGF-β promotes the EMT via the SMAD2/3 pathway, and MMP-9 is a TGF-β target gene that promotes tumor invasion^51–53^. Additionally, in PC3-shC and PC3-shESS2 cells with or without TGF-β treatment, *MMP-9* mRNA was induced in a TGF-β-dependent manner (Fig. 5j), as previously described^53^. Interestingly, TGF-β-dependent MMP-9 mRNA induction in PC3-shESS2 cells was significantly lower than that in PC3-shC cells (Fig. 5j). Moreover, Ess2 significantly enhanced the transcriptional activities of SMAD2/3 (Fig. 5k) and ESS2 knockdown decreased the transcriptional activities of SMAD2/3 (Fig. 5l). These results showed that ESS2 regulated TGF-β expression and the expression of TGF-β target genes by controlling the TGF-β signaling pathway.

We subsequently we examined mRNA levels of ESS2 target genes in CHD1 knockdown PC3 cells (PC3-siCHD1). As shown in Fig. 5m, *TNF* and *TGFB1* were decreased in CHD1 knockdown PC3 cells but expression levels of *VDR* and *PPARG* were not changed. These results show that CHD1 partially regulates mRNA expression levels of ESS2 target genes.

We also compared the expression levels of PARP, histone deacetylase (HDAC), bone morphogenetic protein, C-X-C motif chemokine ligand, and interleukin family genes as well as long noncoding RNAs and HOX genes (Supplementary Fig. 6). However, no significant gene expression changes associated with prostate cancer were observed.

Overall, these results showed that ESS2 mediated several signaling pathways, including CCL2, noncanonical WNT, and TGF-β/SMAD pathways. Such aberrant gene expression in PC3-shESS2 cells may have suppressed prostate cancer progression in xenografts in nude mice.

### *Ess2*^fl/fl^;Rosa26-Cre^ERT2^ mice showed reduced prostate development

**Figure 6:**
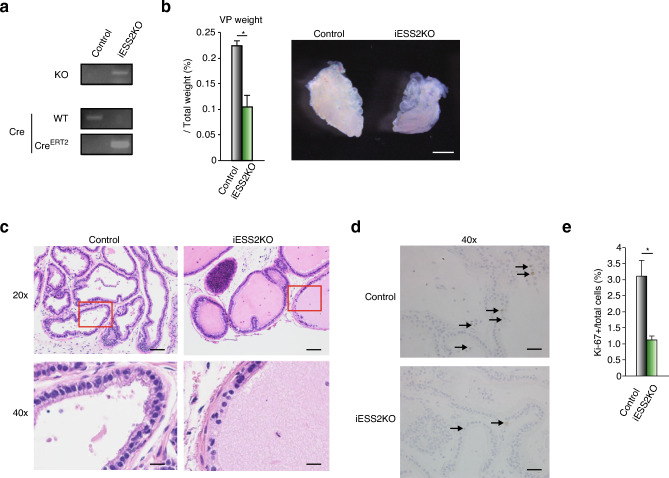
ESS2-knockout mice showed reduced VP development. (**a**) Genotyping PCR of control (Control) and tamoxifen-treated Rosa-CreERT2:Ess2^fl/fl^ (iESS2KO) mice. Primer combinations are described in Supplementary Table. Raw data was shown in Supplementary Fig. 9. (**b**) Left panel: weights of the VP in Control and iESS2KO mice normalized to body weight. n = 3–4. *, *p* < 0.05, Student’s t-test. Right panel: representative VPs in Control and iESS2KO mice. Scale bar = 1 mm. (**c**) HE staining of the VP in Control and iESS2KO mice. Scale bar = 100 μm (20 ×) and 20 μm (40 ×). (**d**) Representative Ki-67 staining of the VP in Control and iESS2KO mice. The sections were counterstained with hematoxylin. The levels of Ki-67-positive cells decreased in iESSKO mice. Scale bar = 50 μm. (**e**) Graph of Ki-67-positive cells / total cells from the VP in Control and iESS2KO mice. Five to six independent areas were counted for each of three mice, and the results are presented as means ± standard deviations. *, *p* < 0.05, Student’s t-test.

We found that ESS2 regulated PC3 cell proliferation by controlling the expression levels of prostate cancer-related genes. However, the functions of ESS2 in prostate development are still unclear. Rodent prostates display ductal branching organization and secretory production specific to each lobe. Prostate epithelial ductal cells are composed of columinal secretory cells lining the lumen and flat basal cells underneath the lumen. In addition, few neuro-endocrine cells are dispersed throughout the gland. To investigate the role of ESS2 in prostate organogenesis, we generated tamoxifen-inducible ESS2-knockout mice (*Ess2*^fl/fl^;Rosa26-Cre^ERT2^) by crossing Rosa26ERT2-Cre mice with ESS2^fl/fl^ mice (RBRC09771, RIKEN) and then treated the mice with tamoxifen. iESS2KO *Ess2*^fl/fl^;Rosa26-Cre^ERT2^ mice (14–15-week-old males) administered tamoxifen did not show lethality and exhibited normal growth (Fig. 6a). The ventral prostate (VP) of *Ess2*^fl/fl^;Rosa26-Cre^ERT2^ (iESS2KO) mice was significantly smaller than that of control mice (Fig. 6b). Moreover, hypoplasia of the anterior prostate (AP) and dorsolateral prostate (DLP) was also observed in iESS2KO mice (data not shown). Hematoxylin and eosin staining of paraffin-embedded prostate tissue sections showed aberrant morphology of ducts consisting of cuboidal luminal cells in iESS2KO mice, whereas control mice showed a single layer of tall columnar luminal cells (Fig. 6c). Moreover, Ki-67 staining of paraffin-embedded prostate tissue sections showed reduced proliferation of VP cells in iESS2KO mice (Fig. 6d,e). These results indicated that ESS2 was a key regulator of prostate development.

## Discussion

**Figure 7:**
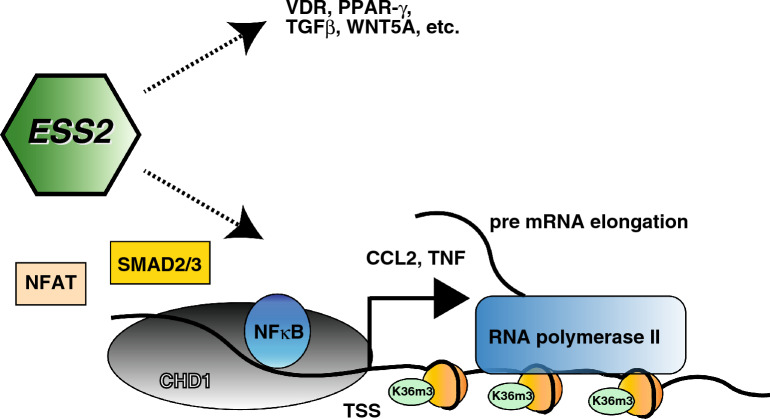
Proposed function of ESS2 in prostate cancer. ESS2 regulates the recruitment of NFκB/CHD1 complex on target promoter and regulates transcription elongation. ESS2 also regulates mRNA expression levels of other genes such as NRs, WNT5A and TGF-β.

In this study, we found that ESS2 knockdown in PC3 cells strongly suppressed tumorigenesis and markedly blocked the expression of NF-κB/CHD1 pathway genes, including *VDR*, *PPAR-γ*, *CCL2*, *WNT5A*, and *TGF-β1* (Fig. 7). These results indicated that ESS2 regulated multiple signaling pathways through genome-wide modulation of mRNA expression in prostate cancer. Recent comprehensive genome-wide analyses have revealed that prostate cancer treatment requires identification of gene mutations, elucidation of the expression profile of each individual, and tailoring the treatment to the individual^7,8^. Our study revealed that the expression levels of some cancer-related genes were correlated with ESS2 expression in patients with prostate cancer. Elucidation of the effects of ESS2 on the expression of prostate cancer-related genes is necessary. In addition, ESS2 knockdown resulted in smaller nuclei; therefore, ESS2 may affect nucleus structure or may regulate mRNA levels of nuclear structure-related genes.

The *ESS2* gene was first cloned as an expression sequence tag located in the 22q11.2 locus, which is related to 22q11.2 deletion syndrome (22q11DS; also known as DiGeorge syndrome or CATCH 22 syndrome)^54^. 22q11DS is associated with various symptoms, such as congenital heart disease, palate abnormalities, immune system dysfunction, and thymic hypoplasia^55^. 22q11.2 deletion syndrome may also be associated with the risk of malignancy^56^; however, the role of ESS2 in patients with cancer is still unclear.

Interestingly, PC3-shESS2 cells showed decreased NF-κB/CHD1 recruitment to target gene promoters. This dysregulation in PC3-shESS2 cells may explain the reduced proliferation of PC3-shESS2 cells. The CHD family consists of nine members, i.e., CHD1–9, which share chromatin organizing (Chromo) domains that bind specifically to modified histones and an SNF2-like ATP-dependent helicase domain that facilitates nucleosome mobilization. CHD1 binds to histone H3K4me3 and H3K36me3 and controls transcriptional elongation^57^. CHD1-knockout mice exhibit embryonic lethality at E5.5 owing to growth retardation^58^. Moreover, CHD1 is involved in cancer progression via regulation of NF-κB target genes in PTEN-deficient prostate cancer^22^, and CHD1 alters androgen receptor binding sites in CHD1-deleted androgen receptor-positive prostate cancer cells^59^ and abrogates anti-androgenic effects by causing induction of transcription factors such as the glucocorticoid receptor^60^. Thus, our results showed that ESS2 regulated the functions of various transcription factors through recruitment of chromatin remodeling factors, such as CHD1.

In recent studies, the relationships between epigenetic factors and carcinogenesis have been clarified, and clinical trials have been conducted for drugs targeting DNA methyltransferase, HDAC, histone acetyltransferase, histone demethylase, and bromodomain and extra-terminal motif in prostate cancer^61^. However, we did not observe any significant changes in the expression of these gene families in PC3-shESS2 cells. Although we examined changes in other histone modifications (H3K4me3, H3K9me3) on the promoters of the *TNF* and *CCL2* genes by ChIP, we could not detect any clear differences. Because ESS2 regulates transcriptional activity, ESS2 may modulate the recruitment or activities of epigenetic factors. However, ESS2 may also regulate lncRNA binding and splicing, and further studies are required.

Previously, we found that ESS2 interacts with another chromatin remodeling factor, BAZ1B^12^. In addition to transcriptional regulation, BAZ1B regulates DNA recombination, replication, and repair^15^. Thus, ESS2 may mediate the recruitment of chromatin remodeling factors, such as BAZ1B and CHD1. However, the molecular mechanisms through which ESS2 is associated with NF-κB/CHD1 still remain unclear. Since ESS2 knockdown in 68–41 murine T-lymphocyte-related cells abrogates the interaction between RORγ/γt and long noncoding RNAs (lncRNAs)^19^, ESS2 may mediate the interaction between lncRNA and transcription factors/chromatin remodeling factors. We examined RNA immunoprecipitation assay on NF-kB and some lnc RNAs, but PC3-shESS2 cells did not show abrogation of the interaction (data not shown). Further studies are required to elucidate the molecular mechanisms of the ESS2 and NF-κB/CHD1 pathway. Moreover, ESS2 may regulate splicing and DNA repair in cancer cells through its involvement in the splicing C complex^18^, CHD1^62^ and BAZ1B^15^. Additional work is required to elucidate the mechanisms involved in this process.

ESS2 regulates cancer progression, PPAR-γ and VDR responses, and EMT signaling in PC3 cells. Both PPAR-γ and VDR are expressed in various organs and play pivotal roles in cell proliferation, differentiation, and metabolism^35,63^. However, naïve CD4^+^ T-cells in CD4-specific ESS2-knockout mice do not show changes in the mRNA expression levels of these genes. Thus, ESS2 alters the expression of genes in a cell context-dependent manner. Further studies are needed to assess these molecular mechanisms. One possibility is that ESS2 depletion may alter the genome-wide recruitment of chromatin remodeling factors, such as CHD1, resulting in dysregulation of transcription. Previous ChIP-seq data^22^ have revealed that CHD1 also binds to the *PPARγ* and *WNT5A* promoters. Thus, ESS2-dependent alterations in CHD1 recruitment can regulate genome-wide gene expression in cancer.

We found that TGF-β-dependent induction of MMP-9^53^ was abrogated in PC3-shESS2 cells. Our studies showed, for the first time, that ESS2 may regulate the EMT via the TGF-β1 signaling pathway. Because MMP-9 expression levels were reduced in PC3-shESS2 cells following TGF-β treatment, intracellular molecules involved in the TGF-β pathway, such as SMAD2/3, may be regulated by ESS2.

ESS2 also plays critical roles in prostate development. Although many transcription factors and signaling pathways are critical for prostate development^64^, it is unclear which signaling pathways and transcription factors are regulated by ESS2. In this study, we performed RT-qPCR analysis of androgen receptor mRNA in the VP of control and iESS2KO mice; however, significant differences in expression were not found (data not shown). Because luminal cells express high levels of Runx1^65^ and Nkx3.1^66^, ESS2 may regulate the transcriptional activities of these genes. Further studies of the mechanisms of ESS2 in prostate development should be performed.

Our results suggested that ESS2/protein binding inhibitors may suppress cancer proliferation by regulating the structure of chromatin. In particular, small molecule ESS2/CHD1 binding inhibitors may be effective candidates for anticancer agents.

In summary, we demonstrated the roles of ESS2 in prostate cancer progression for the first time. Because ESS2 regulates numerous genes involved in prostate cancer, the discovery of ESS2-regulated molecules may contribute to the development of novel molecular targeted therapies for prostate cancer.

## Methods

### Clinical samples

Clinical samples were conducted in accordance with Declaration of Helsinki provided for human experimentation (1964). This study has been approved by the appropriate institutional review boards of the Institute of Medical Science of the University of Tokyo (reference no. 2021–104-0308). Informed consent was obtained from all subjects involved in the study.

Samples from clinically localized prostate cancer obtained by radical prostatectomy (n = 21) and normal prostate tissues obtained by radical cystectomy (n = 5) were collected from men ages 54–78 years (median, 71 years) and 68–84 years (median, 74 years), respectively, at the University of Tokyo. The average pre-operative serum prostate-specific antigen levels were 10.0 ± 1.62 ng/mL (range, 4.30–29.13 ng/mL) and 3.82 ± 1.50 ng/mL (range, 2.16–8.82 ng/mL), respectively, and Gleason scores in the prostate cancer group were 4 + 3 = 7 (n = 9), 4 + 4 = 8 (n = 5), 4 + 5 = 9 (n = 6), and 5 + 4 = 9 (n = 1).

### RNA isolation from FFPE specimens

Total mRNA was extracted from FFPE specimens using a nucleic acid isolation kit (Ambion, Life Technologies, Grand Island, NY)^67^. FFPE specimens were cut into 11-μm-thick slices. Pathologists distinguished the cancer region of FFPE specimens by hematoxylin and eosin (HE) staining. The cancer region of unstained FFPE specimens was divided using a knife and collected into a microcentrifuge tube. Paraffin was removed using 100% xylene with incubation for 3 min at 50 °C to melt the paraffin. After removal of xylene, the pellet was washed with 100% ethanol twice and dried at room temperature for 45 min. The pellet was digested with 100 μL digestion buffer (Ambion, Life Technologies) and 4 μL protease and incubated for 15 min at 50 °C and then 15 min at 80 °C. A mixture of 120 μL isolation additive (Ambion, Life Technologies) and 275 μL of 100% ethanol was added to the sample, passed through a filter cartridge by centrifuge to isolate nucleic acids, and rinsed. A mixture of 4 μL DNase, 6 μL 10 × DNase buffer, and 50 μL nuclease-free water was added to the cartridge, incubated at room temperature for 30 min, rinsed, and centrifuged. Then, 30 μL nuclease-free water was added, and purified RNA was obtained after centrifugation for 1 min at room temperature.

### Cell culture and chemicals

PC3, CRW22Rv1, DU145, and LNCaP cells were purchased from American Type Culture Collection (Manassas, VA, USA). The cells were cultured in RPMI 1640 medium (Thermo Fisher Scientific) supplemented with 10% fetal bovine serum (FBS; Thermo Fisher Scientific) and 100 U/mL penicillin–streptomycin (Nacalai Tesque Inc.). HEK293 (human embryonic kidney 293) cells were cultured in Dulbecco’s modified Eagle medium containing 10% fetal bovine serum (FBS), 100 unit/mL penicillin, and 100 ug/mL streptomycin. The cells were maintained at 37 °C and 5% CO_2_ in a humid environment. We used 10 ng/mL recombinant TGF-β (PeproTech) for MMP-9 induction. Troglitazone was provided by Prof. S Kato (Iryo Sosei University and Tokiwa Foundation, Japan), and VDR ligand (1α,25-dihydroxyvitamin D_3_) was previously described^68^.

To establish PC3 cells stably expressing ESS2 shRNA, we used SureSilencing shRNA Plasmid for Human ESS2 for Puromycin resistance (cat. no. KH16627P; Qiagen, Valencia, CA, USA). After transfection with ESS2 shRNA plasmids or negative control shRNA plasmid using FuGENE HD (Promega, Madison, WI, USA), cells were cultured with 1 μg/mL puromycin for at least 2 weeks and selected.

For transient ESS2 siRNA transfection experiments, we purchased MISSION esiRNA (Sigma, St. Louis, MO, USA), ESS2 esiRNA (cat. no. EHU001101-20UG) and Control Renilla luc (cat. no. EHURLUC-20UG). After transfection of ESS2 esiRNA or RLUC esiRNA into LNCaP or HEK293 cells using Lipofectamine RNAiMAX (Thermo Fisher Scientific), cells were incubated for 24 h, harvested, and subjected to RNA extraction. For transient CHD1 siRNA transfection experiments, we purchased Silencer™ Select (Thermo Fisher Scientific) CHD1 (s2975 4390824) and Control (4390843). After transfection of CHD1 siRNA or Control siRNA into PC3 cells using Lipofectamine RNAiMAX (Thermo Fisher Scientific), cells were incubated for 72 h, harvested, and subjected to RNA extraction.

### Xenograft experiment

All mouse xenograft experiments were approved by Teikyo University Animal Ethics Committee (19–026) and conformed to the ARRIVE guidelines. The in vivo tumor growth of human prostate cancer cells transduced with a negative control shRNA or ESS2 shRNA was determined using a subcutaneous transplant xenograft model. PC3-shC or PC3-shESS2 cells (1 × 10^6^) in phosphate-buffered saline (PBS)/Matrigel mixture were injected subcutaneously into 5-week-old male nude mice (CLEA Japan Inc.) under deep anesthesia with isoflurane (Pfizer). The resulting tumors were evaluated once a week. Once the largest tumor diameter reached the maximal tumor diameter allowed under our institutional protocol, all mice were killed, and tumors were collected and weighted.

### Plasmids and reagents

NF-κB-RE-luc reporter vector was purchased from Priomega (E8491). AP1 RE-luc reporter vector and dexamethasone were gifts from Dr. H. Ogawa. Expression vectors of NFAT, CA-TGFβR and luciferase reporter vectors for NFAT RE-luc and SBE-luc were donated by Prof. A. Yoshimura^69^. Expression vectors of p65 and p50 were gifts from Dr. S. Sawatsubashi. Ess2 expression vector was used as previously described^12^. Recombinant human TNF-α was purchased from Peprotech (300-01A). Phorbol 12-myristate 13-acetate (PMA) and ionomycin were purchased from Sigma-Aldrich.

### Luciferase reporter assay

For luciferase reporter assays, transfections of HEK293 cells were performed by the calcium phosphate co-precipitation method as described previously ^70^. Briefly, 8 h after transfection, compounds were added. Cells were harvested after 24 h and were assayed for luciferase and β-galactosidase activities using a luminometer and a microplate reader (Molecular Devices, Sunnyvale, CA). Co-transfection experiments used 50 ng of reporter plasmid, 10 ng of pCMX-β-galactosidase and 15 ng of each expression plasmid in each well of a 96-well plate. Luciferase data were normalized to the internal β-galactosidase control and represent the means +/− S.D. All points were performed in triplicate and repeated at least twice in independent experiments.

### Fluorescence observation

Cells were fixed in 4% paraformaldehyde and washed with PBS. Cell specimens were stained with rhodamine phalloidin (Cytoskelton, Inc.) and mounted with DAPI (Vectashield; Vector Laboratories, Burlingame, CA, USA). Mounted cell specimens were analyzed with a confocal microscope (ZSM710; Carl Zeiss).

### BrdU staining and MTT assay

BrdU staining was performed using a BrdU kit (Sigma) according to the manufacturer’s protocol. Cell viability was measured using 3-(4,5-dimethylthiazol-2-yl)-2,5-diphenyltetrazolium bromide (MTT; Nacalai Tesque Inc.) assays after dissolution of MTT in PBS (final concentration, 5 mg/mL). Cells were incubated in 96-well plates at 10,000 cells/well in 100 μL RPMI1640 supplemented with 10% FBS at 37 °C in a 5% CO_2_ incubator. Next, 10 μL MTT reagent was added, and samples were incubated for 4 h. Absorbance was recorded at 570 nm with a FlexStation 3G (Molecular Devices).

### Western blotting

For western blotting and/or ChIP, we used antibodies against p65 (cat. no. 6956 [Cell Signaling Technology, Danvers, MA, USA]; cat. no. ab16502 [Abcam, Cambridge, UK]), PPAR-γ (cat. no. PP-A3409A-00; Perseus Proteomics), VDR (cat. no. sc-13133; Santa Cruz Biotechnology, Santa Cruz, CA, USA), ESS2/DGCR14 (cat. no. HPA001222; Sigma), CHD1 (cat. no. sc-271626; Santa Cruz Biotechnology), p21/CDKN1A (cat. no. sc-817; Santa Cruz Biotechnology), p27/CDKN1B (cat. no. sc-1641; Santa Cruz Biotechnology), GAPDH (cat. no. 10494–1-AP; Protein Group) and β-actin (cat. no. sc-47778; Santa Cruz Biotechnology). Western blotting experiments were performed as previously described^12^ and the original blots were shown in the Supplementary Figs. 7a–c and 8. Proteins recognized by antibodies were revealed by an electrochemiluminescence (ECL) technique, following the Manufacturer's instructions (Amersham Biosciences, Amersham, U.K.). To standardize and quantify the immunoblots, we used the photo documentation system Image Quant LAS 400 mini (GE Healthcare, IL, USA).

### ChIP-qPCR

ChIP assays were performed according to the manufacturer’s protocol (Millipore). Briefly, cells were fixed with 1% formaldehyde, and chromatin was sheared by sonication to 300–500 bp. Chromatin was immunoprecipitated with control IgG or specific antibodies (p65, CHD1, and H3K36Me3 [MABI0333; MBL]) overnight at 4 °C and then incubated with protein A-agarose-salmon sperm DNA (Millipore) for an additional 2 h. After washing and elution, protein-DNA crosslinks were disrupted by heating at 65 °C overnight. Immunoprecipitated DNA was purified with QIAquick spin columns (Qiagen) and analyzed by qPCR with a StepOne (Bio-Rad Laboratories, CA, USA) using Light Cycler SYBR Green I Master Mix (Takara Bio). Relative quantification was performed using the 2^-ΔCT^ method, where ΔCT is the difference between the mean CT value of triplicates of the sample and that of the input control. Primer sequences are shown in Supplementary Table.

### RNA isolation and qRT-PCR

Total RNA was extracted using TRIzol (Invitrogen, Carlsbad, CA, USA), and first-strand cDNA was synthesized from total RNA using PrimeScript Reverse Transcriptase (Takara Bio). For qPCR, a StepOne system (Thermo Fisher Scientific) was used with Light Cycler SYBR Green I Master Mix (Takara). Relative quantification was performed using the 2^-ΔCT^ method, where ΔCT is the difference between the mean CT value of triplicate samples. Primers are listed in the Supplementary Table.

### Microarray and bioinformatic analysis

RNA was extracted with an RNeasy mini kit (Qiagen), and samples were subjected to microarray analysis by Takara Bio (Japan). Microarray data are available in the Gene Expression Omnibus (GSE173998). We analyzed microarray data using GeneSpring (Agilent). GSEA was performed using the GSEA software package (GSEA v2.2.3), and all gene set files were obtained from www.broadinstitute.org/gsea/. To investigate correlations of ESS2 expression with other genes, we used the R2: Genomics Analysis and Visualization Platform (http://r2.amc.nl) and drew graphs by RStudio.

### Generation of *Ess2*^fl/fl^;Rosa26-Cre^ERT2^ mice

All iEss2KO mice-related animal experiments were performed according to the protocols, which adhered to the Nihon University Rules concerning Animal Care and Use, approved by Nihon University Animal Care and Use Committee (AP17M055-1), and conformed to the ARRIVE guidelines. To generate tamoxifen-induced Ess2-knockout (iEssKO) mice, *Ess2*^fl/fl^ mice (RBRC09771, RIKEN BRC) were crossed with Rosa26-Cre^ERT2^ mice (Jax stock #008,463). The genotype was confirmed by extracting DNA and performing PCR with genotyping primers. The original electrophoresis data was shown in the Supplementary Fig. 9. Oligonucleotide sequences are listed in the Supplementary Table.

### Histological analysis

HE staining and K-i67 staining was performed by Genostaff Co., Ltd. (Japan). For immunohistochemical evaluation of ESS2, we used anti-ESS2/DGCR14 antibody (cat. no. HPA001222; Sigma), HRP-conjugated goat anti-rabbit antibody (cat. no. 5220-0336; SeraCare Life Sciences,Inc.), ImmPACT DAB (cat. no. SK-4105; Vector laboratories) and Mayer's Hematoxylin Solution (cat. no. 8656; Sakura Finetek Japan).

### Statistical analyses

Data are presented as means ± standard deviations. Equality of variances was assessed using F-tests. Comparisons between two groups were made using two-tailed Student’s t-tests or two-tailed Welch’s t-tests when the variances were equal or unequal, respectively. *P* values less than 0.05 were considered statistically significant. For GSEA, a false discovery rate q-value less than 0.05 was considered statistically significant.

## Supplementary Information

Supplementary Information.

Supplementary Tables.

Supplementary Figure 1.

Supplementary Figure 2.

Supplementary Figure 3.

Supplementary Figure 4.

Supplementary Figure 5.

Supplementary Figure 6.

Supplementary Figure 7.

Supplementary Figure 8.

Supplementary Figure 9.

## Data Availability

The raw data of microarray are publicly available on the GEO repository; Accession No. GSE173998 (token; ydclmyqkzbadbkb). Ess2^fl/fl^ mice (RBRC09771) were registered with the RIKEN BRC. All remaining data are contained within this article and the supporting information.

## References

[1] Siegel RL, Miller KD, Jemal A (2019). Cancer statistics, 2019. CA Cancer J. Clin..

[2] Zhang D (2020). Intron retention is a hallmark and spliceosome represents a therapeutic vulnerability in aggressive prostate cancer. Nat. Commun..

[3] de Wit R (2019). Cabazitaxel versus abiraterone or enzalutamide in metastatic prostate cancer. N. Engl. J. Med..

[4] Martin GA, Chen AH, Parikh K (2017). A novel use of olaparib for the treatment of metastatic castration-recurrent prostate cancer. Pharmacotherapy.

[5] de Bono J (2020). Olaparib for metastatic castration-resistant prostate cancer. N. Engl. J. Med..

[6] LeVee A (2021). Clinical utility of olaparib in the treatment of metastatic castration-resistant prostate cancer: a review of current evidence and patient selection. Oncol. Targets Ther..

[7] Thomas G (2008). Multiple loci identified in a genome-wide association study of prostate cancer. Nat. Genet..

[8] Miyamoto DT (2015). RNA-Seq of single prostate CTCs implicates noncanonical Wnt signaling in antiandrogen resistance. Science.

[9] Rosenfeld MG, Lunyak VV, Glass CK (2006). Sensors and signals: a coactivator/corepressor/epigenetic code for integrating signal-dependent programs of transcriptional response. Genes Dev..

[10] Clapier CR, Iwasa J, Cairns BR, Peterson CL (2017). Mechanisms of action and regulation of ATP-dependent chromatin-remodelling complexes. Nat. Rev. Mol. Cell Biol..

[11] Mills AA (2017). The chromodomain helicase dna-binding chromatin remodelers: family traits that protect from and promote cancer. Cold Spring Harb. Perspect. Med..

[12] Takada I (2015). DGCR14 induces Il17a gene expression through the RORgamma/BAZ1B/RSKS2 complex. Mol Cell Biol.

[13] Ivanov II (2006). The orphan nuclear receptor RORgammat directs the differentiation program of proinflammatory IL-17+ T helper cells. Cell.

[14] Wang J (2016). ROR-gamma drives androgen receptor expression and represents a therapeutic target in castration-resistant prostate cancer. Nat. Med..

[15] Sharif SB, Zamani N, Chadwick BP (2021). BAZ1B the protean protein. Genes (Basel).

[16] Clark DE (2005). The serine/threonine protein kinase, p90 ribosomal S6 kinase, is an important regulator of prostate cancer cell proliferation. Cancer Res..

[17] Cronin R, Brooke GN, Prischi F (2021). The role of the p90 ribosomal S6 kinase family in prostate cancer progression and therapy resistance. Oncogene.

[18] Hegele A (2012). Dynamic protein-protein interaction wiring of the human spliceosome. Mol. Cell.

[19] Takada I (2018). Ess2 bridges transcriptional regulators and spliceosomal complexes via distinct interacting domains. Biochem. Biophys. Res. Commun..

[20] Takada, I. H. S., Takahashi, S., Yanaka, K., Ogawa, H., Tsuchiya, M., Yokoyama, A., Sato, S., Ochi, H., Nakagawa, T., Kobayashi, T., Nakagawa, S., Makishima, M. Transcriptional coregulator Ess2 controls survival of post-thymic CD4+ T cells through the Myc and IL-7 signaling pathways. *J. Biol. Chem.* (2022). 10.1016/j.jbc.2022.102342 PMC9436822 35933014

[21] Tai S (2011). PC3 is a cell line characteristic of prostatic small cell carcinoma. Prostate.

[22] Zhao D (2017). Synthetic essentiality of chromatin remodelling factor CHD1 in PTEN-deficient cancer. Nature.

[23] Tsai EY, Jain J, Pesavento PA, Rao A, Goldfeld AE (1996). Tumor necrosis factor alpha gene regulation in activated T cells involves ATF-2/Jun and NFATp. Mol. Cell Biol..

[24] Fatica A, Bozzoni I (2014). Long non-coding RNAs: New players in cell differentiation and development. Nat. Rev. Genet..

[25] Salameh A (2015). PRUNE2 is a human prostate cancer suppressor regulated by the intronic long noncoding RNA PCA3. Proc. Natl. Acad. Sci. USA.

[26] Diamond MS, Farzan M (2013). The broad-spectrum antiviral functions of IFIT and IFITM proteins. Nat. Rev. Immunol..

[27] Liu X (2019). IFITM3 promotes bone metastasis of prostate cancer cells by mediating activation of the TGF-beta signaling pathway. Cell Death Dis..

[28] Tan H (2015). Effects of interferons and double-stranded RNA on human prostate cancer cell apoptosis. Oncotarget.

[29] Mangelsdorf DJ (1995). The nuclear receptor superfamily: The second decade. Cell.

[30] Lazar MA (2017). Maturing of the nuclear receptor family. J. Clin. Invest..

[31] Mazaira GI (2018). The nuclear receptor field: a historical overview and future challenges. Nucl. Receptor Res..

[32] Shiota M, Fujimoto N, Kashiwagi E, Eto M (2019). The role of nuclear receptors in prostate cancer. Cells.

[33] Zheng Y (2017). Loss of the vitamin D receptor in human breast and prostate cancers strongly induces cell apoptosis through downregulation of Wnt/beta-catenin signaling. Bone Res..

[34] Tontonoz P, Spiegelman BM (2008). Fat and beyond: The diverse biology of PPARgamma. Annu. Rev. Biochem..

[35] Takada I, Makishima M (2015). PPARgamma ligands and their therapeutic applications: A patent review (2008–2014). Expert Opin. Ther. Pat..

[36] Takada I, Makishima M (2020). Peroxisome proliferator-activated receptor agonists and antagonists: A patent review (2014-present). Expert Opin. Ther. Pat..

[37] Lee NJ (2013). 4-O-methylhonokiol, a PPARgamma agonist, inhibits prostate tumour growth: p21-mediated suppression of NF-kappaB activity. Br. J. Pharmacol..

[38] Radhakrishnan SK, Gartel AL (2005). The PPAR-gamma agonist pioglitazone post-transcriptionally induces p21 in PC3 prostate cancer but not in other cell lines. Cell Cycle.

[39] Gombart AF, Borregaard N, Koeffler HP (2005). Human cathelicidin antimicrobial peptide (CAMP) gene is a direct target of the vitamin D receptor and is strongly up-regulated in myeloid cells by 1,25-dihydroxyvitamin D3. FASEB J..

[40] Wang JY, Swami S, Krishnan AV, Feldman D (2012). Combination of calcitriol and dietary soy exhibits enhanced anticancer activity and increased hypercalcemic toxicity in a mouse xenograft model of prostate cancer. Prostate.

[41] Singh R, Lillard JW, Singh S (2011). Chemokines: Key players in cancer progression and metastasis. Front. Biosci. (Schol Ed).

[42] Jin J (2021). CCL2: An important mediator between tumor cells and host cells in tumor microenvironment. Front. Oncol..

[43] Loberg RD (2006). CCL2 is a potent regulator of prostate cancer cell migration and proliferation. Neoplasia.

[44] Roca H, Varsos Z, Pienta KJ (2008). CCL2 protects prostate cancer PC3 cells from autophagic death via phosphatidylinositol 3-kinase/AKT-dependent survivin up-regulation. J. Biol. Chem..

[45] Murillo-Garzon V, Kypta R (2017). WNT signalling in prostate cancer. Nat. Rev. Urol..

[46] Yamamoto H (2010). Wnt5a signaling is involved in the aggressiveness of prostate cancer and expression of metalloproteinase. Oncogene.

[47] Takahashi S (2011). Noncanonical Wnt signaling mediates androgen-dependent tumor growth in a mouse model of prostate cancer. Proc. Natl. Acad. Sci. USA.

[48] Chen CL (2013). Single-cell analysis of circulating tumor cells identifies cumulative expression patterns of EMT-related genes in metastatic prostate cancer. Prostate.

[49] Ribatti D, Tamma R, Annese T (2020). Epithelial-mesenchymal transition in cancer: a historical overview. Transl Oncol.

[50] Wang YA, Sfakianos J, Tewari AK, Cordon-Cardo C, Kyprianou N (2020). Molecular tracing of prostate cancer lethality. Oncogene.

[51] Konrad L, Scheiber JA, Schwarz L, Schrader AJ, Hofmann R (2009). TGF-beta1 and TGF-beta2 strongly enhance the secretion of plasminogen activator inhibitor-1 and matrix metalloproteinase-9 of the human prostate cancer cell line PC-3. Regul. Pept..

[52] Shiota M (2012). Clusterin mediates TGF-beta-induced epithelial-mesenchymal transition and metastasis via Twist1 in prostate cancer cells. Cancer Res.

[53] Kainuma M, Takada I, Makishima M, Sano K (2018). Farnesoid X receptor activation enhances transforming growth factor beta-induced epithelial-mesenchymal transition in hepatocellular carcinoma cells. Int J Mol Sci..

[54] Lindsay EA (1996). A transcription map in the CATCH22 critical region: identification, mapping, and ordering of four novel transcripts expressed in heart. Genomics.

[55] McLean-Tooke A, Spickett GP, Gennery AR (2007). Immunodeficiency and autoimmunity in 22q112 deletion syndrome. Scand. J. Immunol..

[56] Lambert MP (2018). The 22q11.2 deletion syndrome: Cancer predisposition, platelet abnormalities and cytopenias. Am. J. Med. Genet. A.

[57] Lee Y, Park D, Iyer VR (2017). The ATP-dependent chromatin remodeler Chd1 is recruited by transcription elongation factors and maintains H3K4me3/H3K36me3 domains at actively transcribed and spliced genes. Nucleic Acids Res..

[58] Guzman-Ayala M (2015). Chd1 is essential for the high transcriptional output and rapid growth of the mouse epiblast. Development.

[59] Augello MA (2019). CHD1 loss alters AR binding at lineage-specific enhancers and modulates distinct transcriptional programs to drive prostate tumorigenesis. Cancer Cell.

[60] Zhang Z (2020). Loss of CHD1 promotes heterogeneous mechanisms of resistance to AR-targeted therapy via chromatin dysregulation. Cancer Cell.

[61] Liao Y, Xu K (2019). Epigenetic regulation of prostate cancer: the theories and the clinical implications. Asian J. Androl..

[62] Kari V (2016). Loss of CHD1 causes DNA repair defects and enhances prostate cancer therapeutic responsiveness. EMBO Rep..

[63] Takada I, Makishima M (2015). Therapeutic application of vitamin D receptor ligands: an updated patent review. Expert. Opin. Ther. Pat..

[64] Francis JC, Swain A (2018). Prostate organogenesis. Cold Spring Harb. Perspect. Med..

[65] Mevel R (2020). RUNX1 marks a luminal castration-resistant lineage established at the onset of prostate development. Elife.

[66] Xie Q, Wang ZA (2017). Transcriptional regulation of the Nkx3.1 gene in prostate luminal stem cell specification and cancer initiation via its 3' genomic region. J. Biol. Chem..

[67] Takahashi S (2015). Nanowire analysis of cancer-testis antigens as biomarkers of aggressive prostate cancer. Urology.

[68] Ishizawa M (2008). Lithocholic acid derivatives act as selective vitamin D receptor modulators without inducing hypercalcemia. J. Lipid. Res..

[69] Ichiyama K (2008). Foxp3 inhibits RORgammat-mediated IL-17A mRNA transcription through direct interaction with RORgammat. J. Biol. Chem..

[70] Kaneko E (2003). Induction of intestinal ATP-binding cassette transporters by a phytosterol-derived liver X receptor agonist. J. Biol. Chem..

