# Supplementary material for: ESS2 controls prostate cancer progression through recruitment of chromodomain helicase DNA binding protein 1
Source: Sci Rep. 2023 Jul 31;13:12355. doi: 10.1038/s41598-023-39626-0 (PMC10390525; doi:10.1038/s41598-023-39626-0)
Supplement: Supplementary file 1 — Supplementary Information. [file 41598_2023_39626_MOESM1_ESM.pdf]

Supplementary Information

Fluorescent immunostaining

Immunostaining was performed as previously described [1]. Briefly, cells were fixed in 4% paraformaldehyde and blocked in 5% skim milk/phosphate-buffered saline with 0.1% Tween-20 (PBST). After washing in PBST, cell samples were incubated with mouse anti-ESS2 antibodies [2] in 5% skim milk/PBST, washed with PBST twice, and incubated with Alexa546-conjugated anti-mouse IgG antibodies (Thermo Fisher Scientific, Waltham, MA, USA). Cell specimens were also stained with DAPI (Vectashield with DAPI; Vector Laboratories, Inc., Burlingame, CA, USA). Mounted cell specimens were analyzed with a confocal microscope (ZSM710; Carl Zeiss, Jena, Germany).

References

1. Kainuma, M., Takada, I., Makishima, M. & Sano, K. Farnesoid X Receptor Activation Enhances Transforming Growth Factor beta-Induced Epithelial-Mesenchymal Transition in Hepatocellular Carcinoma Cells. *Int J Mol Sci* **19**, doi:10.3390/ijms19071898 (2018).
2. Takada, I. *et al.* Ess2 bridges transcriptional regulators and spliceosomal complexes via distinct interacting domains. *Biochem Biophys Res Commun* **497**, 597-604, doi:10.1016/j.bbrc.2018.02.110 (2018).
