# Supplementary material for: ESS2 controls prostate cancer progression through recruitment of chromodomain helicase DNA binding protein 1
Source: Sci Rep. 2023 Jul 31;13:12355. doi: 10.1038/s41598-023-39626-0 (PMC10390525; doi:10.1038/s41598-023-39626-0)
Supplement: Supplementary file 2 — Supplementary Tables. [file 41598_2023_39626_MOESM2_ESM.pdf]

Oligonucleotides used for ChIP assay

hCCL2 promoter Fw	5'-CCCCTGCTTCCCTTTCCTAC-3'
hCCL2 promoter Rv	5'-CCATGAGTGATAAGTGGGCTGTT-3'
hCCL2 3'UTR Fw	5'-TACAGAGACTTGGGGAAATTGCTT-3'
hCCL2 3'UTR Rv	5'-GATTCTTGCAAAGACCCTCAAAAC-3'
hTNF 3'UTR Fw	5'-GTCTAAACAATGCTGATTTGGTGAC-3'
hTNF 3'UTR Rv	5'-CCACTGAATAGTAGGGCGATTACA-3'
hTNF TSS Fw	5'-GCTCATGGGTTTCTCCACCA-3'
hTNF TSS Rv	5'-GCCAACAACTGCCTTTATATGTCC-3'

Oligonucleotides used for RT-qPCR

GAPDH FW	5'-ACTTCGCTCAGACACCATGG-3'
GAPDH RV	5'-GTAGTTGAGGTCAATGAAGGG-3'
hESS2 Fw	5'-TGAGAGTTGAAGGGTCGGAAA-3'
hESS2 Rv	5'-TTCTTGGCCCGGTTCTTG-3'
CDKN1A FW	5'-AGACTCTCAGGGTCGAAAAC-3'
CDKN1A RV	5'-TAGGGCTTCCTCTTGGAGAA-3'
CDKN1B FW	5'-AGAGTTAACCCGGGACTTGGA-3'
CDKN1B RV	5'-TGTAGTAGAACTCGGGCAAGCTG-3'
hTNF Fw	5'-TGCTTGTTCCTCAGCCTCTT-3'
hTNF Rv	5'-TGAGGTACAGGCCCTCTGAT-3'
hIL6 Fw	5'-CCTCCAGAACAGATTTGAGAGT-3'
hIL6 Rv	5'-GGTTCTGTGCCTGCAGCTT-3'
hTMPRSS2 Fw	5'-GATCGTGATTTTGTGGTTGTGAG-3'
hTMPRSS2 Rv	5'-AGTTGACCCCGCAGGCTAT-3'
hPPARD Fw	5'-TGCGGCCATCATTCTGTGT-3'
hPPARD Rv	5'-TCTTGATCCGCTGCATCATC-3'
hPPARG Fw	5'-CGTGGATCTCTCCGTAATGGA-3'
hPPARG Rv	5'-AATAAGGTGGAGATGCAGGCTC-3'
hVDR Fw	5'-TACCAGGATTCAGAGACCTCACC-3'
hVDR Rv	5'-GACTCATTGGAGCGCAACAT-3'
hNR4A1 Fw	5'-GATGTGGAGCCTCTCCTTTCC-3'
hNR4A1 Rv	5'-TTTCGTAGCCTCCGCCACT-3'
hNR4A2 Fw	5'-GGGCAGAGCCACATAAACAAA-3'
hNR4A2 Rv	5'-CGGCTGGACAGGCAAAA-3'
hNR4A3 Fw	5'-ATTTGCAGAGCCTGAACCTTG-3'
hNR4A3 Rv	5'-GCTCTTCGACTCTCTTTGGTTCTT-3'
RCRC FW	5'-CAAGACTCATCGCCAAAGCA-3'
RORC RV	5'-CTTTCCACATGCTGGCTACAC-3'
hCYP24A1 Fw	5'-TGAACGTTGGCTTCAGGAGAA-3'
hCYP24A1 Rv	5'-AGGGTGCCTGAGTGTAGCATCT-3'
hCAMP Fw	5'-GCTAACCTCTACCGCCTCCT-3'
hCAMP Rv	5'-GGTCACTGTCCCCATACACC-3'
hCCND1 Fw	5'-TGCCAACCTCCTCAACGAC-3'
hCCND1 Rv	5'-GGCTCTTTTTCACGGGCTC-3'
hCCL2 Fw	5'-CTGCTCATAGCAGCCACCTT-3'
hCCL2 Rv	5'-CAGATCTCCTTGGCCACAAT-3'
hWNT5A Fw	5'-CAAATCCACCAACCCACTAACTC-3'
hWNT5A Rv	5'-TGATCTCCAGCTCCTCCTCTCT-3'
hTGFB1 Fw	5'-CAACAATTCCTGGCGATACCTC-3'
hTGFB1 Rv	5'-AAAGCCCTCAATTTCCCCTC-3'
hMMP9 Fw	5'-CCCTATGTACCGCTTCACTGAG-3'
hMMP9 Rv	5'-AGGGCGACCATAGAGGT-3'
hCHD1 Fw	5'-TGGTGGTCCTCTGGAAAGATTAG-3'
hCHD1 Rv	5'-TCCCAGTCGTCTAAGGTCTGTTT-3'

Oligonucleatides for mouse genotyping

Cre(WT) Fw	5'-CTGGCTTCTGAGGACCG-3'
Cre(WT) Rv	5'-CCGAAAATCTGTGGGAAGTC-3'
Cre(CREERT2) Fw	5'-CGTGATCTGCAACTCCAGTC-3'
Cre(CREERT2) Rv	5'-AGGCAAATTTTGGTGTACGG-3'
KO Fw (5'flox Fw)	5'-ATTTGTGGCCACCACTCATCC-3'
KO Rv (3'flox Rv)	5'-AGTTCCTGGTCTGACTCTGTGAC-3'
