# Supplementary material for: ESS2 controls prostate cancer progression through recruitment of chromodomain helicase DNA binding protein 1
Source: Sci Rep. 2023 Jul 31;13:12355. doi: 10.1038/s41598-023-39626-0 (PMC10390525; doi:10.1038/s41598-023-39626-0)
Supplement: Supplementary file 3 — Supplementary Figure 1. [file 41598_2023_39626_MOESM3_ESM.pdf]

Supplementary Figure 1

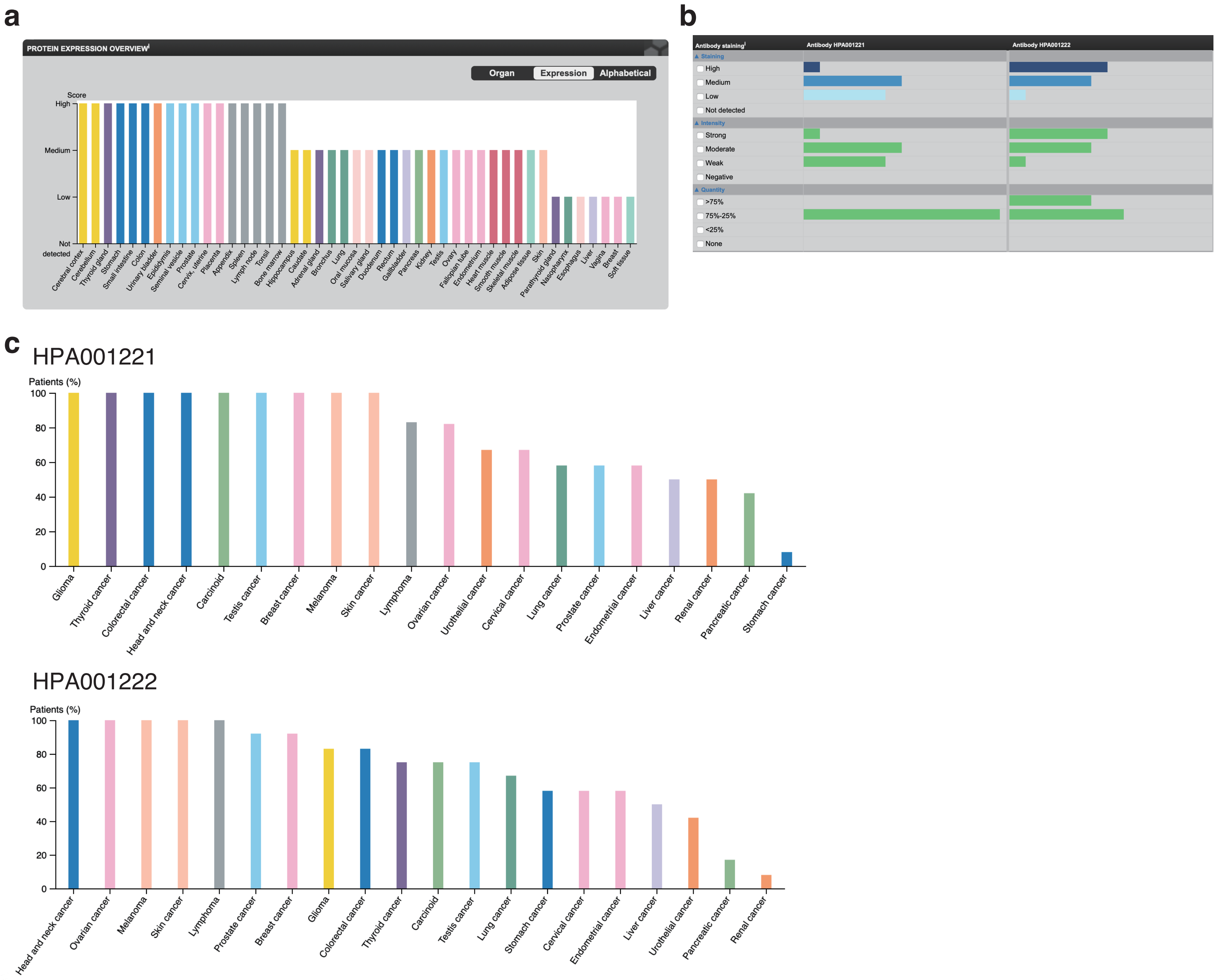

Supplementary Figure 1: (**a**) Normal tissue expression of ESS2 in humans. (**b**) ESS2 expression levels in prostate cancer cells. ESS2 was expressed in all prostate cancer cell lines. (**c**) ESS2 expression levels in cancer tissues, as detected using anti-ESS2 antibodies (HPA001221 or HPA001222). These data were obtained from human protein atlas (https://www.proteinatlas.org/ENSG00000100056-ESS2/pathology/prostate+cancer#Quantity).
