# Supplementary material for: ESS2 controls prostate cancer progression through recruitment of chromodomain helicase DNA binding protein 1
Source: Sci Rep. 2023 Jul 31;13:12355. doi: 10.1038/s41598-023-39626-0 (PMC10390525; doi:10.1038/s41598-023-39626-0)
Supplement: Supplementary file 4 — Supplementary Figure 2. [file 41598_2023_39626_MOESM4_ESM.pdf]

Supplementary Figure 2

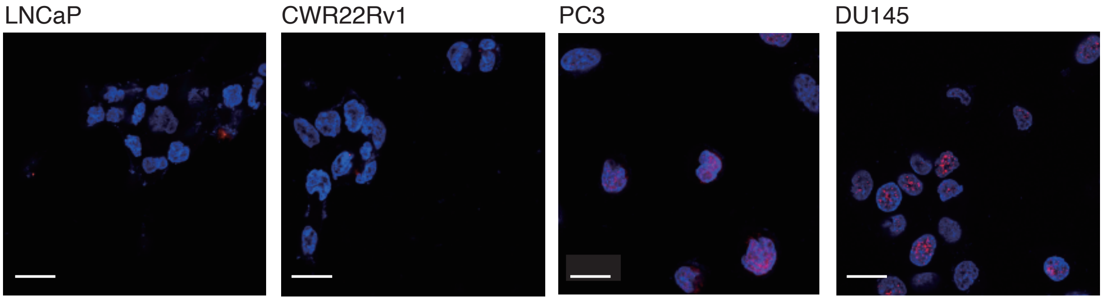

Supplementary Figure 2: Immunofluorescence images obtained using anti-ESS2 antibodies in prostate cancer cell lines. After immunostaining with anti-ESS2 antibodies (red) in each cell line, cells were observed using confocal microscopy. Nuclei are stained with DAPI (blue). Bar scale = 50 μm.
