# Supplementary material for: ESS2 controls prostate cancer progression through recruitment of chromodomain helicase DNA binding protein 1
Source: Sci Rep. 2023 Jul 31;13:12355. doi: 10.1038/s41598-023-39626-0 (PMC10390525; doi:10.1038/s41598-023-39626-0)
Supplement: Supplementary file 5 — Supplementary Figure 3. [file 41598_2023_39626_MOESM5_ESM.pdf]

Supplementary Figure 3

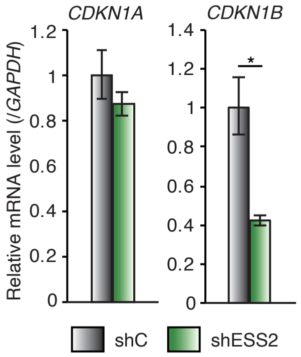

Supplementary Figure 3: RT-qPCR of *CDKN1A* and *CDKN1B* in PC3-shC and PC3-shESS2 cells normalized to the level of *GAPDH* mRNA.
