# Supplementary material for: ESS2 controls prostate cancer progression through recruitment of chromodomain helicase DNA binding protein 1
Source: Sci Rep. 2023 Jul 31;13:12355. doi: 10.1038/s41598-023-39626-0 (PMC10390525; doi:10.1038/s41598-023-39626-0)
Supplement: Supplementary file 6 — Supplementary Figure 4. [file 41598_2023_39626_MOESM6_ESM.pdf]

Supplementary Figure 4

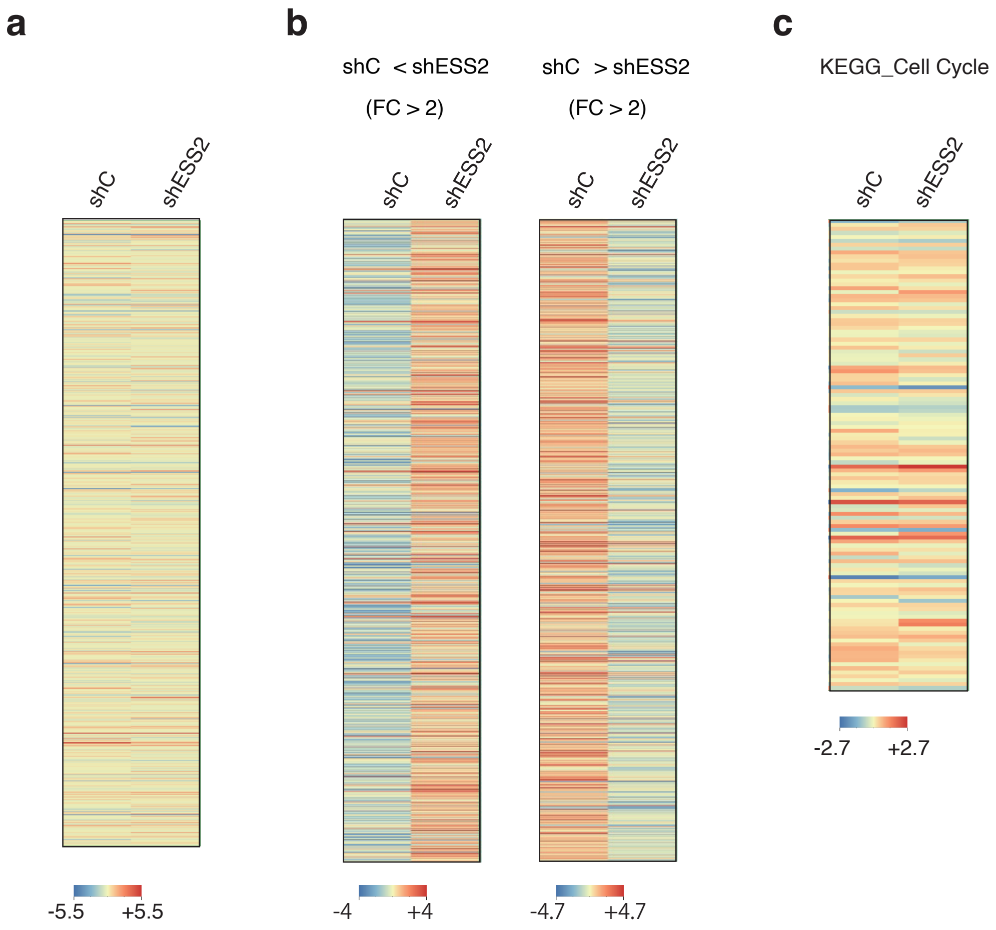

Supplementary Figure 4: Microarray analysis of PC3-shC and PC3-shESS2 cells (Data related to Fig. 2). (**a**) Heatmap analysis of whole gene mRNA in PC3-shC and PC3-shESS2 cells. (**b**) Heatmap of gene mRNA groups with more than 2-fold differences in expression in PC3-shC and PC3-shESS2 cells. The left panel shows overexpressed in PC3-shESS2 cells and the right panel shows downregulation in PC3-shESS2 cells. (**c**) Heatmap of expression in cell cycle-retlated gene sets defined by KEGG.
