# Supplementary material for: ESS2 controls prostate cancer progression through recruitment of chromodomain helicase DNA binding protein 1
Source: Sci Rep. 2023 Jul 31;13:12355. doi: 10.1038/s41598-023-39626-0 (PMC10390525; doi:10.1038/s41598-023-39626-0)
Supplement: Supplementary file 7 — Supplementary Figure 5. [file 41598_2023_39626_MOESM7_ESM.pdf]

Supplementary Figure 5

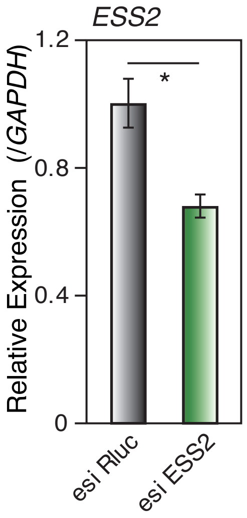

Supplementary Figure 5: RT-qPCR of ESS2 in HEK293 cells transfected with control renilla luciferase esiRNA (esi Rluc) or ESS2 esiRNA (esi ESS2). *, $p < 0.05$.
