# Supplementary material for: ESS2 controls prostate cancer progression through recruitment of chromodomain helicase DNA binding protein 1
Source: Sci Rep. 2023 Jul 31;13:12355. doi: 10.1038/s41598-023-39626-0 (PMC10390525; doi:10.1038/s41598-023-39626-0)
Supplement: Supplementary file 8 — Supplementary Figure 6. [file 41598_2023_39626_MOESM8_ESM.pdf]

Supplementary Figure 6

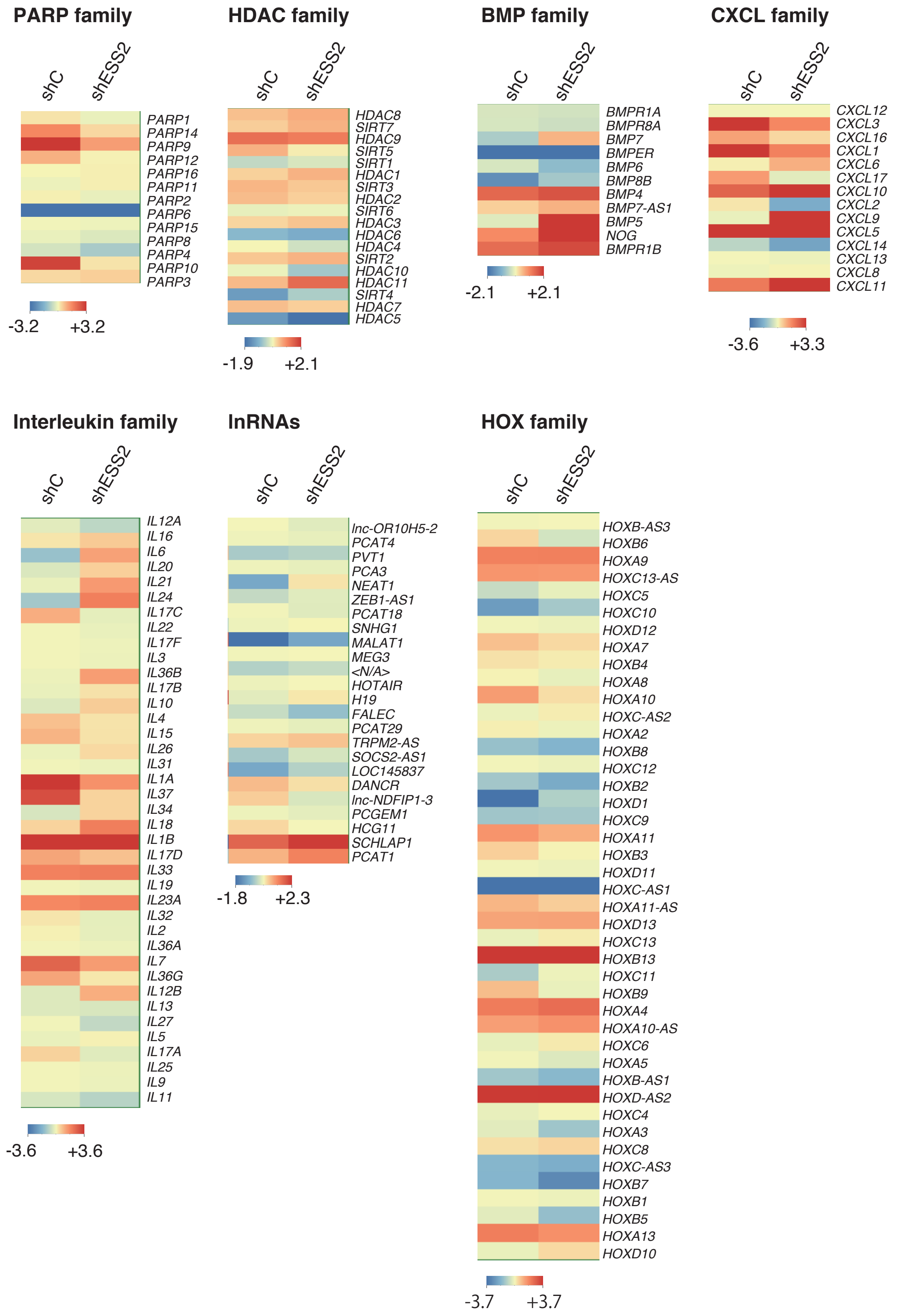

Supplementary Figure 6: Microarray analysis of PC3-shC and PC3-shESS2 cells. Heatmap anaysis of gene expression related to prostate cancer in PC3-shC and PC3-shESS2 cells.
