# Supplementary material for: ESS2 controls prostate cancer progression through recruitment of chromodomain helicase DNA binding protein 1
Source: Sci Rep. 2023 Jul 31;13:12355. doi: 10.1038/s41598-023-39626-0 (PMC10390525; doi:10.1038/s41598-023-39626-0)
Supplement: Supplementary file 9 — Supplementary Figure 7. [file 41598_2023_39626_MOESM9_ESM.pdf]

Supplementary Figure 7

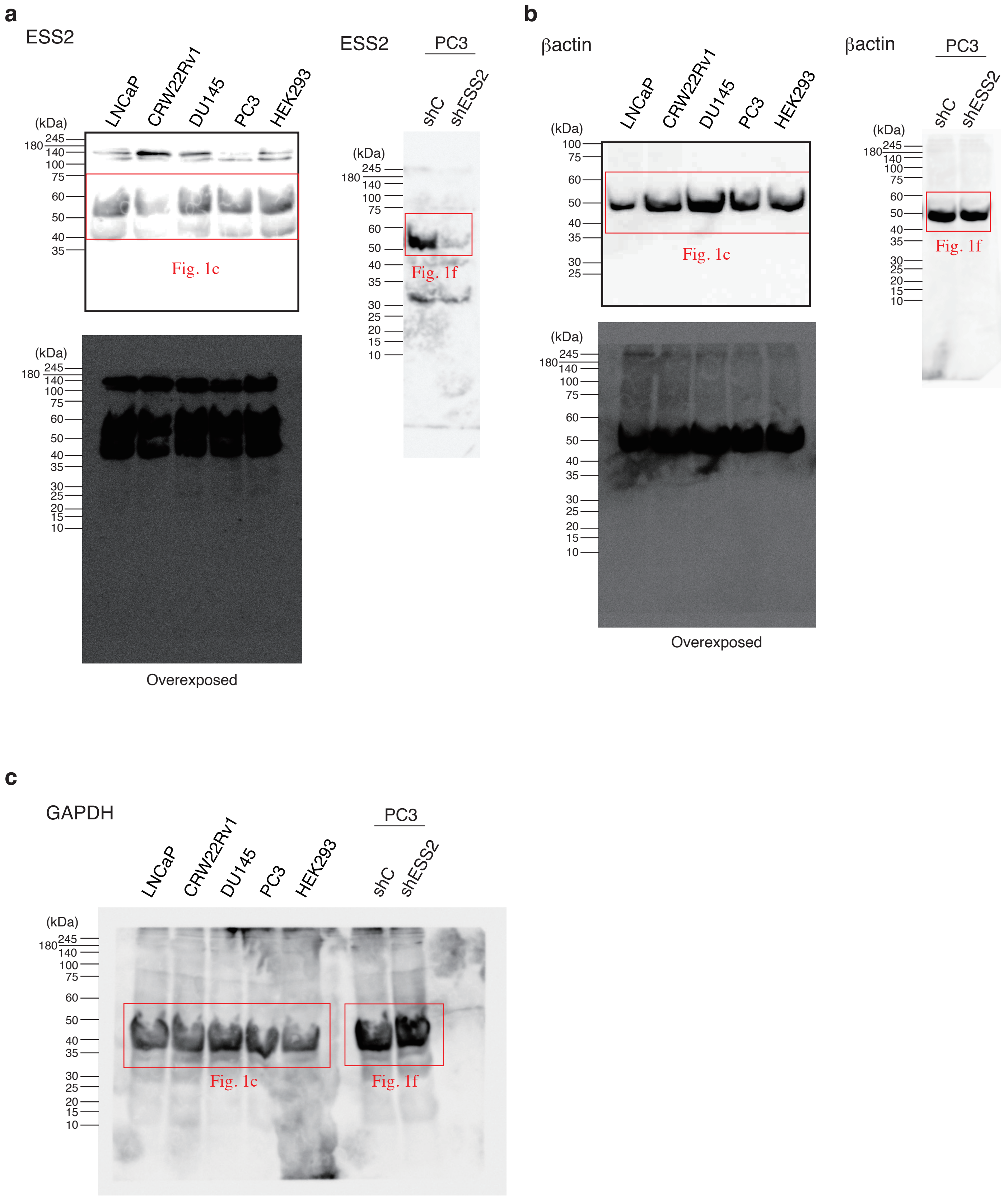

Supplementary Figure 7: Raw data of Western blotting of ESS2 (**a**), βactin (**b**) and GAPDH (**c**) as demonstrated in Fig. 1c and 1f. Some data show overexposed images.
