# Supplementary material for: ESS2 controls prostate cancer progression through recruitment of chromodomain helicase DNA binding protein 1
Source: Sci Rep. 2023 Jul 31;13:12355. doi: 10.1038/s41598-023-39626-0 (PMC10390525; doi:10.1038/s41598-023-39626-0)
Supplement: Supplementary file 10 — Supplementary Figure 8. [file 41598_2023_39626_MOESM10_ESM.pdf]

Supplementary Figure 8

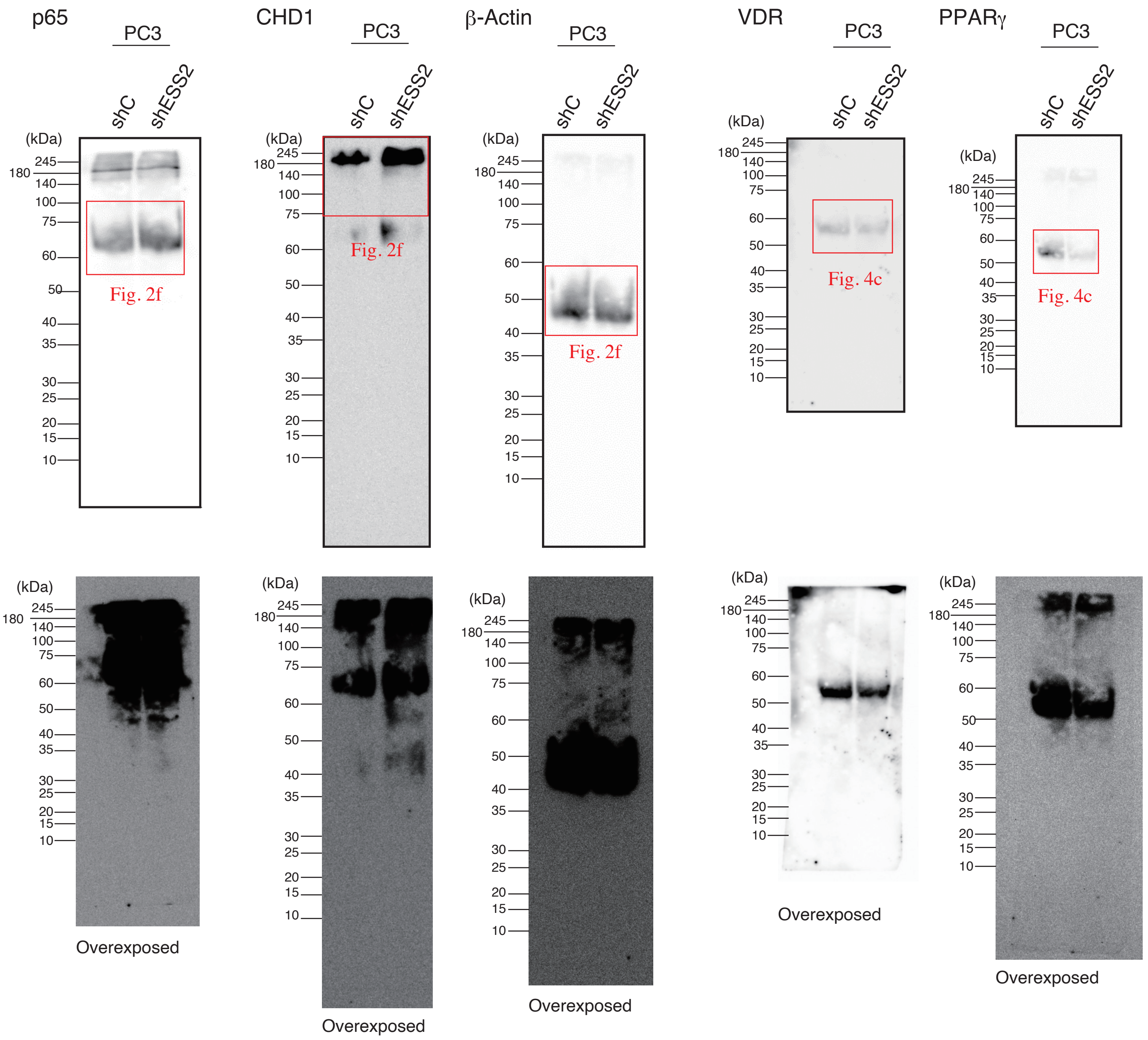

Supplementary Figure 8: Raw data of Western blottings as demonstarated in Fig. 2f and 4c. Overexposed images are shown in low panels.
