# Supplementary material for: ESS2 controls prostate cancer progression through recruitment of chromodomain helicase DNA binding protein 1
Source: Sci Rep. 2023 Jul 31;13:12355. doi: 10.1038/s41598-023-39626-0 (PMC10390525; doi:10.1038/s41598-023-39626-0)
Supplement: Supplementary file 11 — Supplementary Figure 9. [file 41598_2023_39626_MOESM11_ESM.pdf]

Supplementary Figure 9

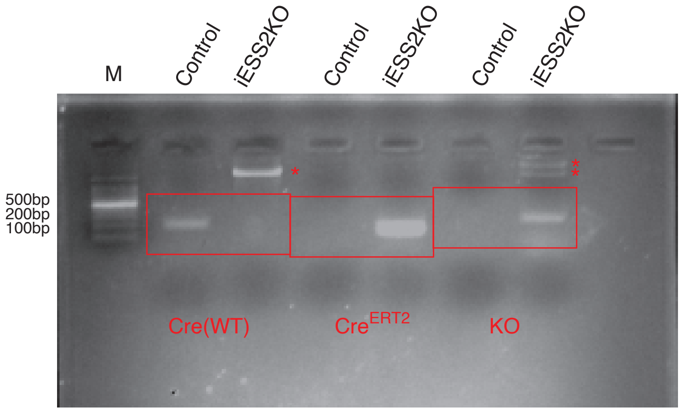

Supplementary Figure 9: Raw data of genotyping PCR of iESS KO mice as demonstrated in Fig. 6a. M shows 100bp DNA ladder (Takara, Japan) and * shows non-specific band.
